# A new mamenchisaurid sauropod from the Lower Phu Kradung Formation, Upper Jurassic of northeastern Thailand

**DOI:** 10.1038/s41598-026-49822-3

**Published:** 2026-07-08

**Authors:** Apirut Nilpanapan, Sita Manitkoon, Varavudh Suteethorn, Komsorn Lauprasert

**Affiliations:** 1 Department of Biology, Faculty of Science, Mahasarakham University, Khamrieng, 44150 Maha Sarakham Thailand; 2 Palaeontological Research and Education Centre, Mahasarakham University, Khamrieng, 44150 Maha Sarakham Thailand; 3 Vertebrate Palaeontology and Evolution Research Unit, Excellence Centre in Evolution of Life, Basin Studies and Applied Palaeontology, Mahasarakham University, Khamrieng, 44150 Maha Sarakham Thailand; 4 Khon Kaen Geopark Association, Khon Kaen, 40150 Thailand

**Keywords:** Sauropoda, Mamenchisauridae, Upper Jurassic, Phu Kradung Formation, Northeastern Thailand, Evolution, Zoology

## Abstract

Mamenchisauridae is a group of long-necked non-neosauropodan eusauropod dinosaurs that were abundant in East Asia during the Middle to Late Jurassic, but their diversity and geographic distribution outside China remain poorly documented. Here we describe *Uragasaurus kalasinensis* gen. et sp. nov., a new sauropod dinosaur from the Phu Kradung Formation of northeastern Thailand. The new taxon is based on a well-preserved anterior dorsal vertebra exhibiting a distinctive combination of characters, including a unique Y-shaped configuration formed by the intraprezygapophyseal and single intraprezygapophyseal laminae and a camellate internal pneumatic structure within the centrum revealed by computed tomography (CT). Phylogenetic analyses recover the new taxon as an early-diverging member of Mamenchisauridae. This discovery represents the first formally named mamenchisaurid from Thailand and expands the known geographic distribution of the clade in Southeast Asia. The occurrence of this taxon in the Lower part of the Phu Kradung Formation also contributes to understanding faunal succession within the unit, supports an Upper Jurassic age for the lower part of the formation, and improves understanding of sauropod diversity in Southeast Asia during the Jurassic-Cretaceous transition.

**Supplementary Information:**

The online version contains supplementary material available at 10.1038/s41598-026-49822-3.

## Introduction

Mamenchisauridae^1^ represents the predominant non-neosauropodan eusauropod clade throughout the Middle to Late Jurassic of East Asia. Members of the clade are characterized by extremely elongated cervical vertebrae with highly developed pneumatic structures, and in derived taxa by procoelous anterior caudal vertebrae, distinct them from other eusauropods^2–6^. These features, although convergently evolved in several Cretaceous neosauropod lineages (including Euhelopodidae, Somphospondyli, and Titanosauria^7–10^), have complicated interpretations of mamenchisaurid relationships and contributed to ongoing uncertainty regarding their phylogenetic position among sauropods^11,12^.

The fossil record of mamenchisaurids is most abundant in China, particularly within the Sichuan Basin, where the Middle to Upper Jurassic deposits of the Shaximiao Formation have yielded various well-known taxa, including *Mamenchisaurus constructus*, along with *M. anyuensis, M. hochuanensis*, *M. sanjiangensis*, *M. youngi*, *Omeisaurus maoianus*, and *O. tianfuensis*^1,13–17^. Additional mamenchisaurid taxa came from the overlying Suining Formation, including *M. anyuensis*, *Qijianglong guokr*, and *Tongnanlong zhimingi*^18–20^. Northwestern China has also produced several members of the clade from the Upper Jurassic Shishugou Formation of Xinjiang, such as *Bellusaurus sui*, *Hudiesaurus sinojapanorum*, *Klamelisaurus gobiensis*, *M. sinocanadorum*, and *Xinjiangtitan shanshanesis*^21–25^. Further discoveries from eastern China (*Anhuilong diboensis* and *Huangshanlong anhuiensis*) and southern China (*Jingiella dongxingensis*)^12,26,27^.

Recent discoveries outside China have challenged the traditional view that mamenchisaurids were restricted to East Asia. Fragmentary vertebral remains from the Middle to Upper Jurassic Khlong Min Formation of Krabi Province in southern Thailand^28^ represent the earliest evidence of mamenchisaurids in Southeast Asia. Additional cervicodorsal elements from the Upper Jurassic to Lower Cretaceous Phu Kradung Formation of northeastern Thailand have also been interpreted as possible members of the clade^29^. Furthermore, the reassessment of the caudal vertebrae previously assigned to *Janenschia robusta* from the Late Jurassic Tendaguru Formation, Tanzania, led to *Wamweracaudia keranjei*, demonstrating that the group also occurred in Africa^30^.

Here we describe *Uragasaurus kalasinensis* gen. et sp. nov., therefore the first formally named mamenchisaurid sauropod from northeastern Thailand, based on an isolated anterior dorsal vertebra recovered from the Phu Noi Locality in Kalasin Province, northeastern Thailand. The specimen originates from the Upper Jurassic to Lower Cretaceous Phu Kradung Formation. This discovery expands the known diversity of mamenchisaurid sauropods in Southeast Asia and provides new information on the geographic distribution and evolutionary history of the clade.

### Anatomical abbreviations

Cpol, centropostzygapophyseal lamina; cprl, centroprezygapophyseal lamina; di, diapophysis; ns, neural spine; pa, parapophysis; pcdl, posterior centrodiapophyseal lamina; pl, pleurocoel; pnfo, pneumatic fossa; po, postzygapophysis; podl, postzygodiapophyseal lamina; ppdl, paradiapophyseal lamina; prdl, prezygodiapophyseal lamina; posl, postspinal lamina; prsl, prespinal lamina; spof, spinopostzygapophyseal fossa; spol, spinopostzygapophyseal lamina; sprf, spinoprezygapophyseal fossa; sprl, spinoprezygapophyseal lamina; stprl, single interprezygapophyseal lamina; tp, transverse process; tpol, intrapostzygapophyseal lamina; tprl, intraprezygapophyseal lamina. Fossae and laminae terminology follows Wilson^32^, Wilson et al.^33^, and Wilson Mantilla^34^.

### Institutional abbreviations

CCG, Chengdu University of Technology, Chengdu, China; IVPP, Institute of Vertebrate Paleontology and Paleoanthropology, Beijing, China; PRC, Palaeontological Research and Education Centre, Mahasarakham University, Thailand; ZDM, Zigong Dinosaur Museum, Sichuan, China.

### Other abbreviations

AL, Longchiaoxiang locality, Anyue County, Sichuan, China; CI, Consistency Index; EIW, Extended Implied Weighting; EQW, Equal Weighting; KS, Kalasin; MPT, most parsimonious tree; OTU, operational taxonomic unit; PK, Phu Kradung Formation; PN, Phu Noi Locality, Kalasin Province, Thailand; RI, Retention Index; TBR, Tree Bisection-Reconnection.

## Results

### Geological setting

**Fig. 1:**
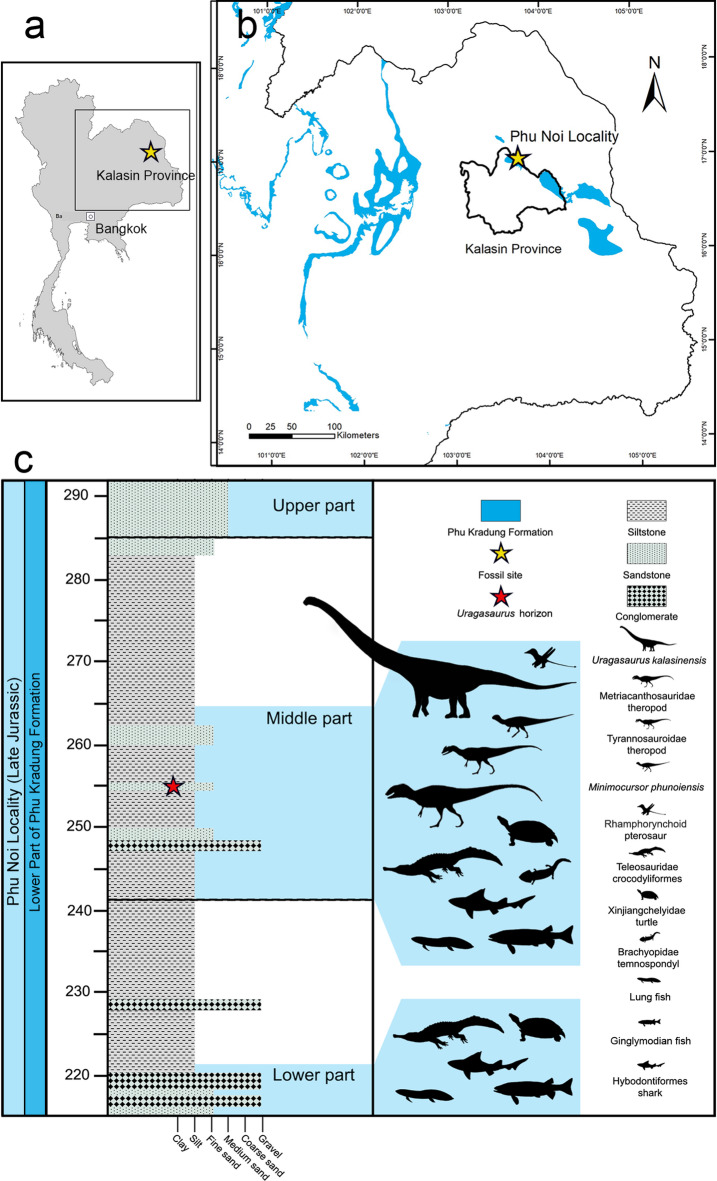
Locality map and section diagram of Phu Noi Locality. The map of Thailand (**a**); The location of Phu Noi Locality and the distribution of Phu Kradung Formation, the northeastern region with the outline of Kalasin Province (edit by Kantanat Trakulveerayut) (**b**); the diagrammatic section of Phu Noi (applied from Chowchuvech et al.^31^) (**c**).

The Phu Noi Locality is situated in Ban Din Chi village, Kham Muang District, Kalasin Province, Northeastern Thailand (Fig. 1b). The site represents one of the most prolific non-marine vertebrate fossil assemblages in Southeast Asia and occurs within the lower part of the Phu Kradung Formation, the basal unit of the Khorat Group^35,36^. The formation consists predominantly of fluvial sandstones, siltstones, and mudstones deposited in a continental basin system, indicating a fluvial depositional environment^35–42^.

The age of the Phu Kradung Formation remains incompletely constrained due to the absence of radiometrically datable horizons. Previous studies have suggested a Late Jurassic age based on regional stratigraphic correlations, vertebrate assemblages, and detrital zircon data^36–44^. However, the chronostratigraphic resolution remains limited, and reliance on single indicators such as the absence of particular palynomorph taxa is insufficient to tightly constrain the age. Consequently, the formation is best regarded as spanning the latest Jurassic and possibly extending into the earliest Cretaceous (Berriasian).

The Phu Noi Locality of the Phu Kradung Formation preserves a complex paleochannel sequence comprising three distinct fossiliferous horizons (Fig. 1c). The lower horizon consists of light grey conglomeratic sandstone representing the paleochannel floor. The middle horizon, situated approximately 10 m above the lower part, comprises brownish-purple and greenish-grey sandy siltstone and mudstone. The upper horizon consists of greyish siltstones within proximal floodplain deposits, located approximately 400 m west-southwest of the middle part^31,36–44^.

The mamenchisaurid specimens described herein were discovered from the middle horizon, which yields diverse vertebrate remains, including isolated and partially articulated skeletal elements. The vertebrate assemblage includes hybodontiformes sharks, ginglymodian fishes, lungfishes, basal eucryptodiran turtles, teleosaurid crocodyliformes, and neornithischian dinosaurs^63,64,35–42,44^. Additional taxa currently under study include brachyopid temnospondyl, rhamphorynchoidea pterosaur, basal tyrannosauroid, and metriacanthosaurid theropods, and the mamenchisaurid sauropod described herein^31,42,43^.

Taken together, the vertebrate assemblage from the Phu Noi locality shows strong biogeographic affinities with Jurassic–earliest Cretaceous faunas from the Junggar, Turpan, and Sichuan basins of China^22,23,45^, supporting placement of the Phu Kradung Formation near the Jurassic–Cretaceous transition, although precise stage-level correlation remains uncertain.

### Systematic palaeontology

Dinosauria Owen^46^

Saurischia Seeley^47^

Sauropodomorpha von Huene^48^

Sauropoda Marsh^49^

Eusauropoda Upchurch^4^

Mamenchisauridae Young & Zhao^1^

*Uragasaurus kalasinensis* gen. et sp. nov.

LSID: urn:lsid.org:act:4AEEEE6B-156F-466D-963A-6B52F018799C.

### Holotype

The isolated anterior dorsal vertebra PRC 460 has been housed at the Palaeontological Research and Education Centre (PRC), Mahasarakham University, Thailand. Following standard paleontological protocols, the vertebrae were mechanically prepared using pneumatic tools and fine brushes to remove the surrounding sediment matrix.

### Associated sauropod materials

KS 34-581. Anterior dorsal neural arch. KS 34-586. Anterior dorsal neural arch. KS 34-587. Left coracoid. KS 34-588. Left fibula. KS 34-602a. Middle cervical vertebra. KS 34-602b. Right cervical rib. KS 34-692. Middle to posterior dorsal vertebra. PN 13-23. Posterior dorsal vertebra.

The holotype specimen (PRC 460) was recovered from a localized excavation area within the same stratigraphic horizon. Although several additional sauropod elements were discovered in close proximity, these remains lack diagnostic features that overlap and cannot be confidently referred to the holotype individual. These specimens are therefore treated as associated sauropod materials rather than elements of the holotype individual. Consequently, the diagnosis of *Uragasaurus kalasinensis* is based solely on the holotype vertebra.

### Etymology

The genus name “Uraga” originates from the Sanskrit word उरग, meaning “snake” or “serpent”, referring to the distinguished long neck of the family. The term “saurus” is derived from the Greek word *saûros,* meaning lizard. The specific epithet refers to Kalasin Province, where the specimens are from.

### Locality

Phu Noi Locality, Kalasin Province, northeastern Thailand (16.93298° N, 103.72327° E).

### Horizon

Lower part of the Phu Kradung Formation, Khorat Group.

### Age

Latest Jurassic, based on regional stratigraphic correlations and the composition of the vertebrate assemblage; precise chronostratigraphic constraints remain uncertain.

### Diagnosis

*Uragasaurus kalasinensis* gen. et sp. nov. is a mamenchisaurid sauropod diagnosed by the following unique combination of characters (autapomorphy is marked by *): (1) anterior dorsal vertebra with prominent, elongated teardrop-shaped pneumatic fossae on the distal portion of the transverse processes*; (2) intraprezygapophyseal laminae (tprl) meeting ventromedially to form a Y-shaped configuration in anterior view, incorporating a single vertical intraprezygapophyseal lamina (stprl); and (3) shallow, subtriangular pleurocoel lacking an internal septum.

### Description and comparisons

The specimen is completely preserved except for heavy anteroposterior compression, which obscures its lateral morphology. The centrum is opisthocoelous, the parapophysis is located on the ventrolateral margin of the neural arch that is close to the dorsolateral margin of the centrum, the dorsally curved transverse processes flare horizontally with a downward diapophysis facet, and the neural spines exhibit a U-shape bifurcation cleft in anterior view, which lacks vertically elongated sagittal prespinal (prsl) and postspinal laminae (posl). PRC 460 is equivalent to the second dorsal vertebra of *M*. *hochuanensis* and *M*. *youngi*, the third dorsal vertebra of *M. anyuensis*, and the fifth dorsal vertebra of *Klamelisaurus*^1,14,18,25^. Comparative measurements of anterior dorsal vertebrae in selected mamenchisaurids are provided in Table 1, supporting the interpretation of the holotype as an anterior dorsal vertebra. Moreover, the internal camellate structure revealed by CT scanning (see Internal pneumatic structure), which is typical of Mamenchisauridae and closely related East Asian forms such as *Omeisaurus*, is presented ^1,14,16,19,24,50–52^.

**Table 1:** Measurements of the anterior dorsal vertebra of *Uragasaurus kalasinensis* (PRC 460) with the 3rd dorsal vertebra of *M. anyuensis* (AL001), the 2nd dorsal vertebra of *M. hochuanensis* (CCG V 20401) and *M. youngi* (ZDM 0083), and the 5th dorsal vertebra of *Klamelisaurus* (IVPP V9492). Italicized measurements indicate dimensions that may be affected by post-depositional compression and should be interpreted cautiously. All measurements are in millimeters.

Taxa	CL	ACH	ACW	PCH	PCW	NAH	NAW	NSH	NSW	H
*Uragasaurus kalasinensis*	*94.9*	*182.3*	*231.6*	*166.2*	*218.9*	*183.5*	*698.7*	*171.4*	*320.3*	*548.9*
*Mamenchisaurus anyuensis*	*–*	*164.3*	*215.82*	*–*	*–*	*–*	*641.79*	*–*	*188.89*	*517.12*
*Mamenchisaurus hochuanensis*	*148.00*	*71.3*	*128.57*	*214.3*	*352.04*	*151.11*	*727.35*	*210.34*	*299.8*	*640.36*
*Mamenchisaurus youngi*	282.14	159.39	195.42	203.97	240.00	129.47	*569.16*	*157.56*	*238.17*	*488.35*
*Klamelisaurus gobiensis*	160	–	–	230	181	–	*–*	*–*	*–*	520

ACH, anterior centrum dorsoventral height; ACW, anterior centrum transverse width; CL, centrum length; H, total height; NAH, neural arch dorsoventral height (from the dorsal margin of centrum to the base of postzygapophyses); NAW, neural arch width; NSH, neural spine dorsoventral height (from the base of postzygapophyses to the summit of neural spine); NSW, neural spine width; PCH, posterior centrum dorsoventral height; PCW, posterior centrum width.

Several disarticulated sauropod elements were recovered within a few meters surrounding the holotype in the assemblage. Because these remains were not found articulated and include elements showing morphology that may differ from the holotype, they are regarded here as associated sauropod materials rather than confidently referred specimens.

### PRC 460 anterior dorsal vertebra

**Fig. 2:**
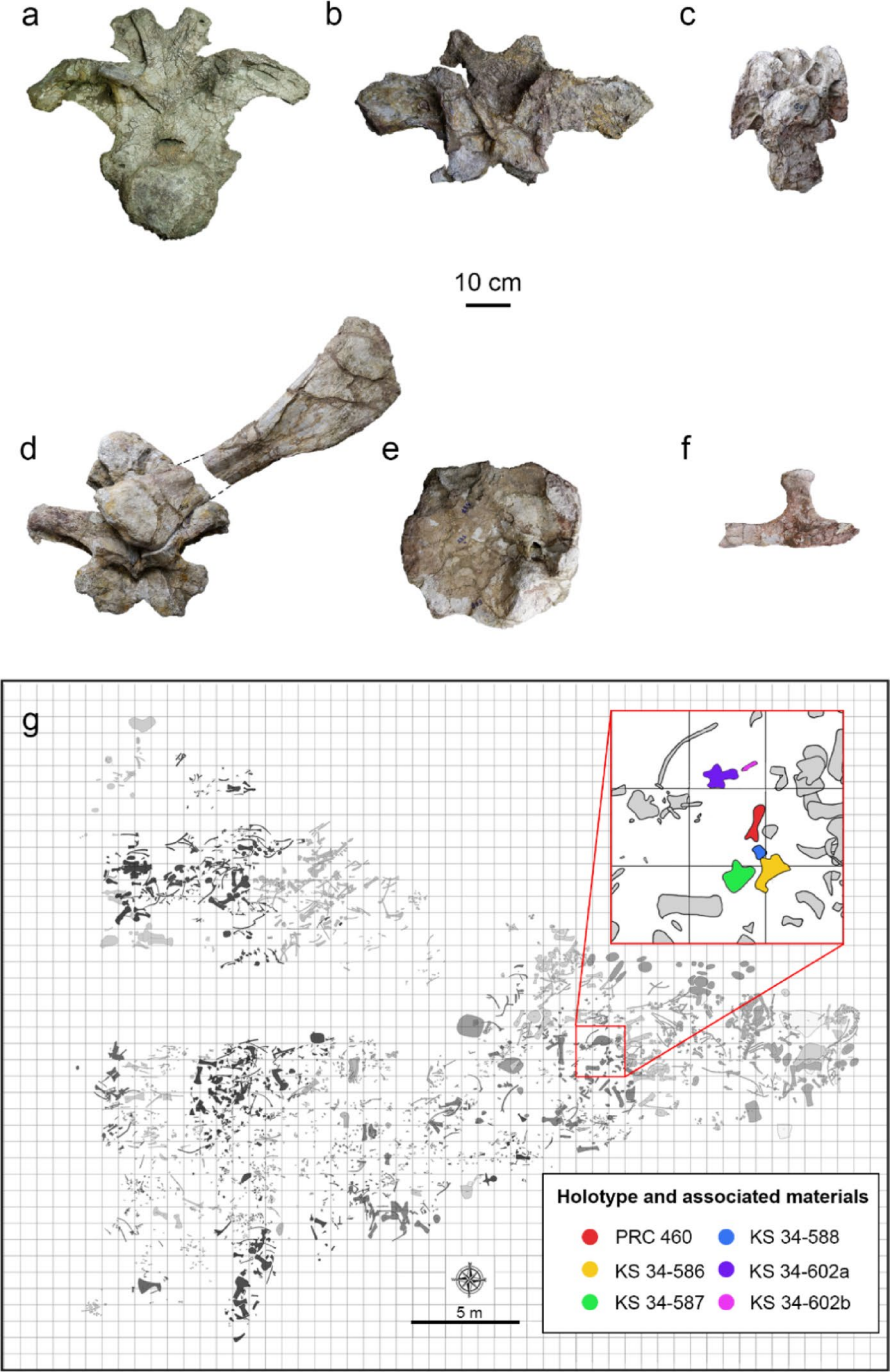
Holotype of *Uragasaurus kalasinensis* (PRC 460) and associated materials in the quarry map. PRC 460 Anterior dorsal vertebra in anterior view (**a**), KS 34-581 anterior dorsal neural arch in anterior view (**b**), KS 34-602a middle cervical vertebra in ventral view (**c**), KS 34-586 anterior dorsal neural arch in anterior view, attached by KS 34-588 fibula (**d**), KS 34-587 coracoid in lateral view (**e**), KS 34-602b right cervical rib in lateral view (**f**). Quarry map showing the spatial distribution of the holotype and associated materials from the Phu Noi Locality (**g**). PRC 460, representing the new taxon *Uragasaurus kalasinensis*, is indicated in red. Associated sauropod elements include KS 34-586, KS 34-587, KS 34-588, and KS 34-602a–b, highlighted in yellow, green, blue, purple, and pink, respectively. The inset shows a close-up of the excavation grid highlighting the relative positions of the holotype and nearby associated materials. Each grid square represents 0.75 × 0.75 m.

**Fig. 3:**
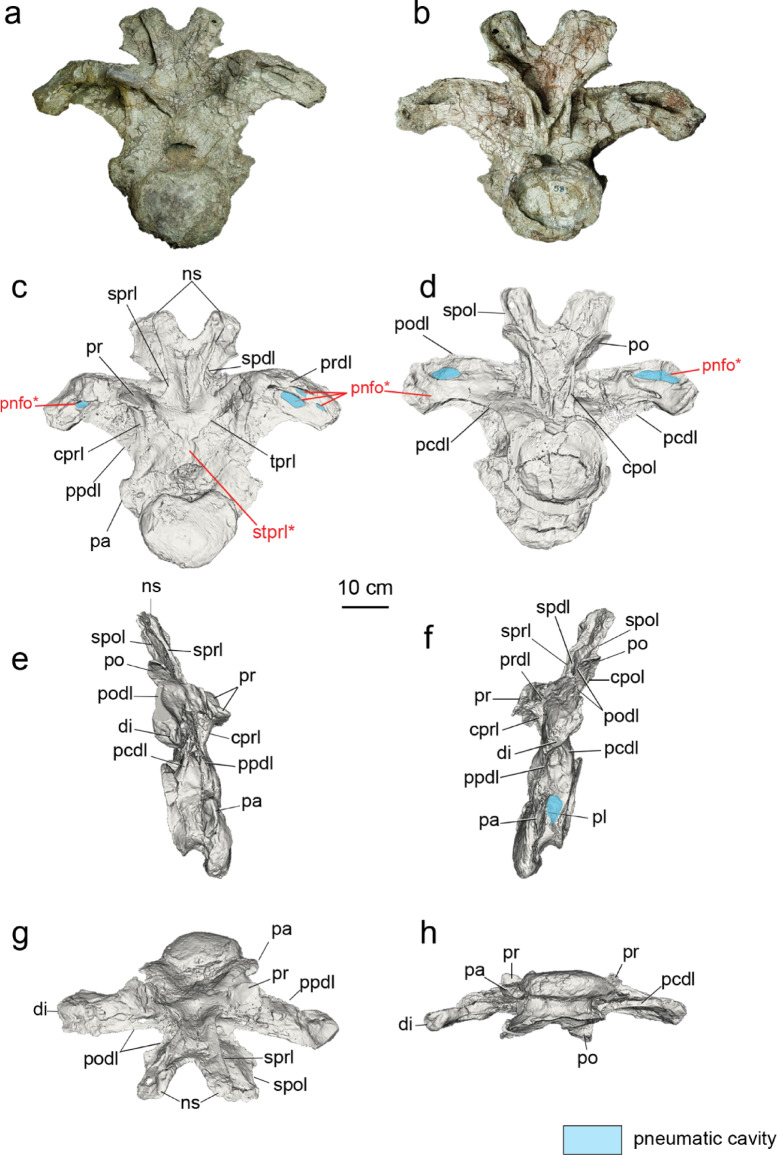
The holotype anterior dorsal vertebra of *Uragasaurus kalasinensis* (PRC 460) in anterior (**a**) and posterior (**b**) views. Digital rendering of the specimen in anterior (**c**), posterior (**d**), right lateral (**e**), left lateral (**f**), dorsal (**g**), and ventral (**h**) views. Asterisk refers to an autapomorphic character. The blue highlight indicates the pneumatic fossa and pleurocoel.

The anterior dorsal vertebra PRC 460 is well-preserved in anteroposterior perspective, representing lamina and fossa architectures (Figs. 2a, b, and 3). The centrum is opisthocoelous with a wider-than-high hemispherical outline, while the neural canal opening is transversely expanded and ellipsoid in outline. The sub-rectangular parapophysis is situated on the dorsolateral margin of the centrum, consistent with anterior dorsal vertebrae of eusauropods. The ventral surface of the centrum is anteroposteriorly concave and transversely convex without a ventral midline keel. The marginal border between the lateral and ventral surfaces is absent. The shallow and indistinct sub-triangular pleurocoel (sensu Britt^51^), which is posteriorly tapering, is situated on the anterodorsal area of the lateral surfaces, and the dorsal margin reaches to the ventral area of the neural arch. Moreover, it lacks excavation of pneumatic foramina inside the pleurocoel, and the internal septum is absent.

The prezygapophysis has large, dorsomedially oriented, transversely ellipsoid articular facets. It is ventrally supported by a pair of robust vertical buttresses of centroprezygapophyseal lamina (cprl), which are situated at two-thirds of the height of the neural arch. The tprl ventromedially connects to the counterpart at the middle of the neural arch, where the dorsal end of the stprl forms a Y-shaped configuration, which is considered the unique morphological feature of *Uragasaurus kalasinensis*. Furthermore, prezygapophysis laterally articulates with the prezygodiapophyseal lamina (prdl), which progressively enhances the dorsoventral thickness toward the distal end, forming the dorsolateral-facing platform of the diapophysis. The platform has a rough surface indicative of m. longissimus dorsi attachment, which is represented in the posterior cervical and anterior dorsal vertebrae of archosaurs^25,51^.

The ventrolaterally directed facet of the diapophysis has a smoothly curved dorsal border, and the transverse process curves upward distally. The postzygodiapophyseal lamina (podl) and dorsolateral-facing platform lines are on the dorsal edge, while the paradiapophyseal lamina (ppdl) lines are on the ventral edge. On the anterior surface of the transverse process, the ventral area beneath the platform contains three pneumatic fossae, one large horizontally elongated and two small, teardrop-shaped, located near the diapophysis. On the posterior surface, a prominent, elongated teardrop-shaped fossa, which is larger than the anterior one, is located on the upper part of the distal area of the transverse process.

The anterior dorsal neural spine is a short U-shaped, bifurcated, and absent of a median tubercle. The anterior surface of the spine is covered with a broad but shallow V-shaped spinoprezygapophyseal fossa (sprf) with many weak V-shaped streaks that line vertically, interpreted as muscle attachment scars, along the sagittal plane. The postzygapophysis and spinopostzygapophyseal lamina (spol) are convexly curved to the ventrolateral margin of the spine, as shown in the anterior view. A large postspinal fossa (posf) is located on the posterior side of the spines, bounded laterally by the spol and ventrally by the intrapostzygapophyseal lamina (tpol). The posf extends ventrally from the shallow plain on the neural spine, passing through the proximal half and ventral boundary of the tpol between the postzygapophyses.

### Internal pneumatic structure

**Fig. 4:**
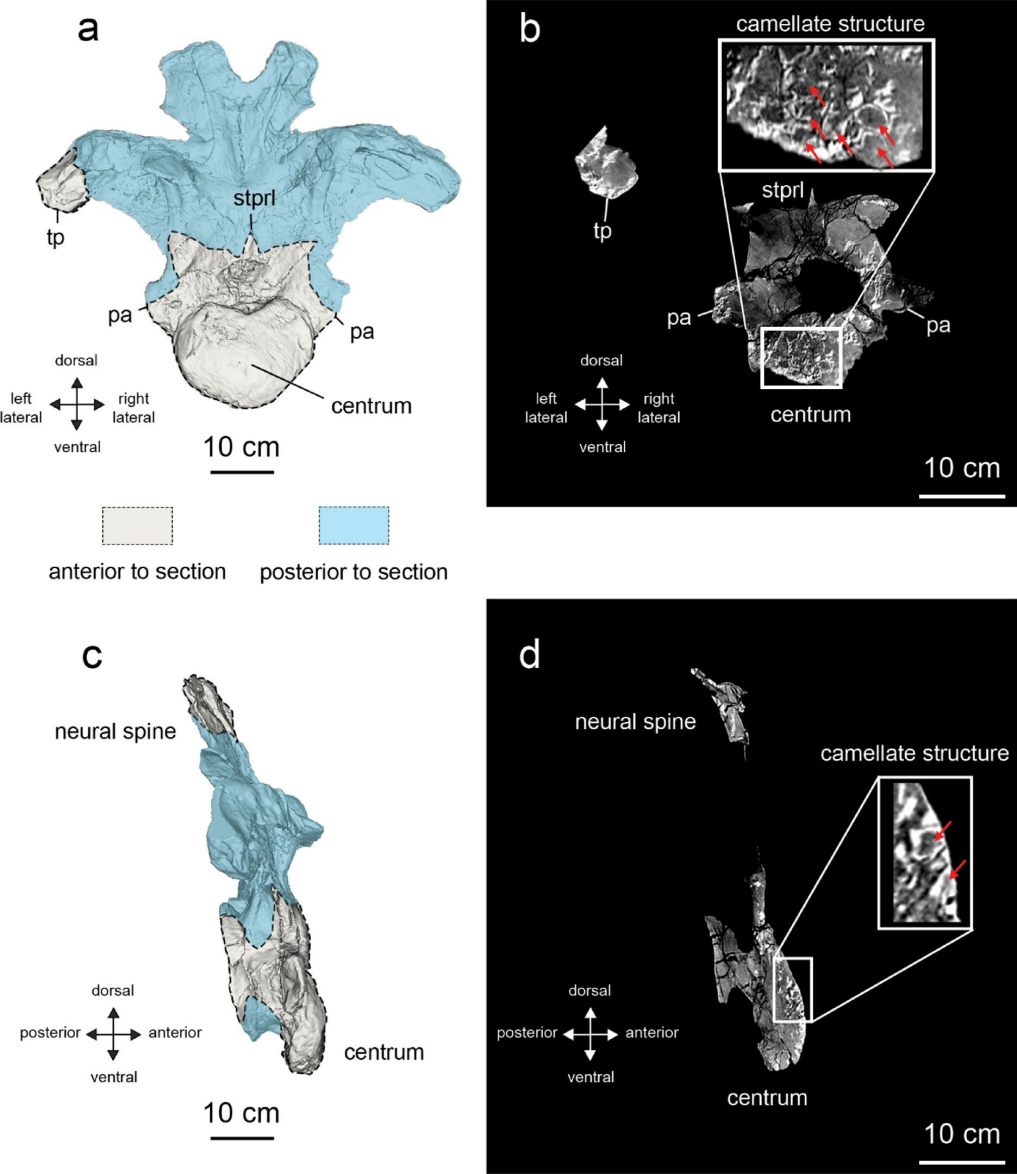
CT scan of the anterior dorsal vertebra of *Uragasaurus kalasinensis* (holotype PRC 460). Three-dimensional reconstructions of the vertebra in anterior view (**a**) and right lateral view (**c**). Corresponding CT sections in anterior view (**b**) and right lateral view (**d**). The section plane corresponds to the dashed line separating the grey (anterior) and blue (posterior) regions in the 3D reconstructions. White boxes highlight camellate pneumatic cavities within the centrum. Red arrows indicate polygonal camellae within the camellate internal structure of the centrum. Black-and-white arrows indicate anatomical orientation in each panel.

Computed tomography (CT) data reveal that the centrum of the anterior dorsal vertebra PRC 460 exhibits a camellate internal pneumatic structure composed of numerous small, irregular chambers separated by thin bony septa (Fig. 4). Quantitative measurements of chamber dimensions were not attempted because the internal cavities are partially obscured by mineral infilling and preservation artifacts. This condition differs from the procamerate internal structure seen in certain neosauropods such as *Haplocanthosaurus*^52^ and the camerate condition found in macronarians and diplodocoids, such as *Camarasaurus* and *Apatosaurus*^52^. This indicates a relatively advanced degree of pneumatic invasion within the vertebral centrum of the mamenchisaurids. Camellate internal pneumaticity is commonly developed on the presacral vertebrae of the derived eusauropods, including mamenchisaurids, but also occurs convergently within Neosauropoda, particularly in Somphospondyli and Titanosauria. Evidence of camellate structure in mamenchisaurids has been documented from broken or eroded vertebrae, such as the first and second dorsal vertebrae of *M. youngi*^14^, the damaged third dorsal vertebra of *M. anyuensis*^18^, the cervicodorsal of Phu Dan Ma taxon^29^, and the ninth dorsal vertebra of *Xinjiangtitan*^24,50^. However, in derived neosauropods such as somphospondylans and titanosaurs, the camellae are typically smaller, more numerous, and separated by thinner bony septa^8,52,53^. The presence of this condition in *Uragasaurus kalasinensis* further supports the interpretation that complex vertebral pneumatic architectures evolved repeatedly among eusauropods and derived sauropod clades.

### Comparisons

The overall laminar architecture, bifurcated neural spine morphology, and configuration of the transverse processes of PRC 460 closely resemble those of the anterior dorsal vertebrae of *M. youngi* and *M. hochuanensis*. For example, the ventral surface of the centrum is anteroposteriorly concave and transversely convex without a ventral midline keel^1,54^, differing from *M. sanjiangensis*, which possesses a ventral keel^17^. The pleurocoel lacks an internal septum and secondary pneumatic excavation like *Jingiella*, *M. anyuensis*, *M. hochuanensis*, and *M. sanjiangensis*^1,12,17,18^, in contrast to the condition observed in the Phu Dan Ma cervicodorsal (KS26-4)^29^. The ventrolaterally directed diapophysis facet resembles those of *Klamelisaurus, O. tianfuensis*, *M. hochuanensis, M. youngi,* and *Xinjiangtitan,* with a smoothly curved dorsal border and an upwardly curved transverse process^1,14,16,24,25,50^. Furthermore, the prezygapophysis has large, dorsomedially oriented, transversely ellipsoid articular facets, as in *M. anyuensis*^18^, *M. hochuanensis*^1^, and *M. youngi*^14^. However, several features distinguish *Uragasaurus kalasinensis* from these and other mamenchisaurids.

The stprl is present only in *Uragasaurus* among mamenchisaurids, but it is also present convergently in the macronarian neosauropod *Europasaurus holgeri*^55^. The formation of the Y-shaped lamina by stprl and tprl is not reported in other mamenchisaurids to date. This configuration differs from the V-shape condition in cervicodorsal of Phu Dan Ma taxon^29^, anterior dorsal vertebrae of *Klamelisaurus*^23^, and *M. youngi*^14^, and the U-shaped configuration in *M. hochuanensis*^1^. On the posterior surface, a prominent, elongated teardrop-shaped fossa, which is larger than the anterior one, is located on the upper part of the distal area of the transverse process, which is present in *Uragasaurus* but absent from other mamenchisaurids and *Omeisaurus*^1,14,18,56^. Finally, the anterior dorsal neural spine is a short U-shaped, bifurcated, and absent of a median tubercle, similar to *Klamelisaurus*^21,25^ but different from the V-shaped bifurcated neural spines of *M. anyuensis*, *M. hochuanensis*, and *M. youngi*^1,14,18^.

### Associated sauropod materials

Several additional sauropod elements were recovered in close proximity to the holotype (PRC 460). However, these fossils cannot be confidently assigned to the same individual because of the taphonomic complexity and aggregation of the assemblage. These include: (1) a poorly preserved anterior dorsal neural arch fragment catalogued under the same field number as the holotype (KS 34-581); (2) an isolated anterior dorsal neural arch (KS 34-586) that bears an attached distal fibula fragment (KS34-588); (3) a separate fibula shaft corresponding to the piece attached to KS34-586 (KS34-588); (4) a dorsoventrally compressed cervical vertebra (KS34-602a), which is dorsally attached with the indeterminates rib shaft fragment; (5) an incomplete cervical rib fragment (KS34-602b); and (6) a fragmentary coracoid of indeterminate laterality (KS34-587). Because these specimens lack overlapping diagnostic characters with the holotype, none of them is included in the diagnosis of *Uragasaurus kalasinensis*.

Moreover, additional dorsal vertebra (PN 692) was recovered from the same excavation area but is located at a considerable distance from the holotype on the bone map and lacks clear articulation or size correspondence. The last specimen is a posterior dorsal vertebra (PN 13-23) recovered from an unknown grid area of the locality, and it uses the unusual Identical abbreviation and number because of the reformation of the collection ID of PRC since 2013. These specimens are therefore not considered part of the holotype and are treated as associated sauropod materials. Detailed anatomical descriptions of these associated materials are provided in the Supplementary Information.

### Phylogenetic analysis

**Fig. 5:**
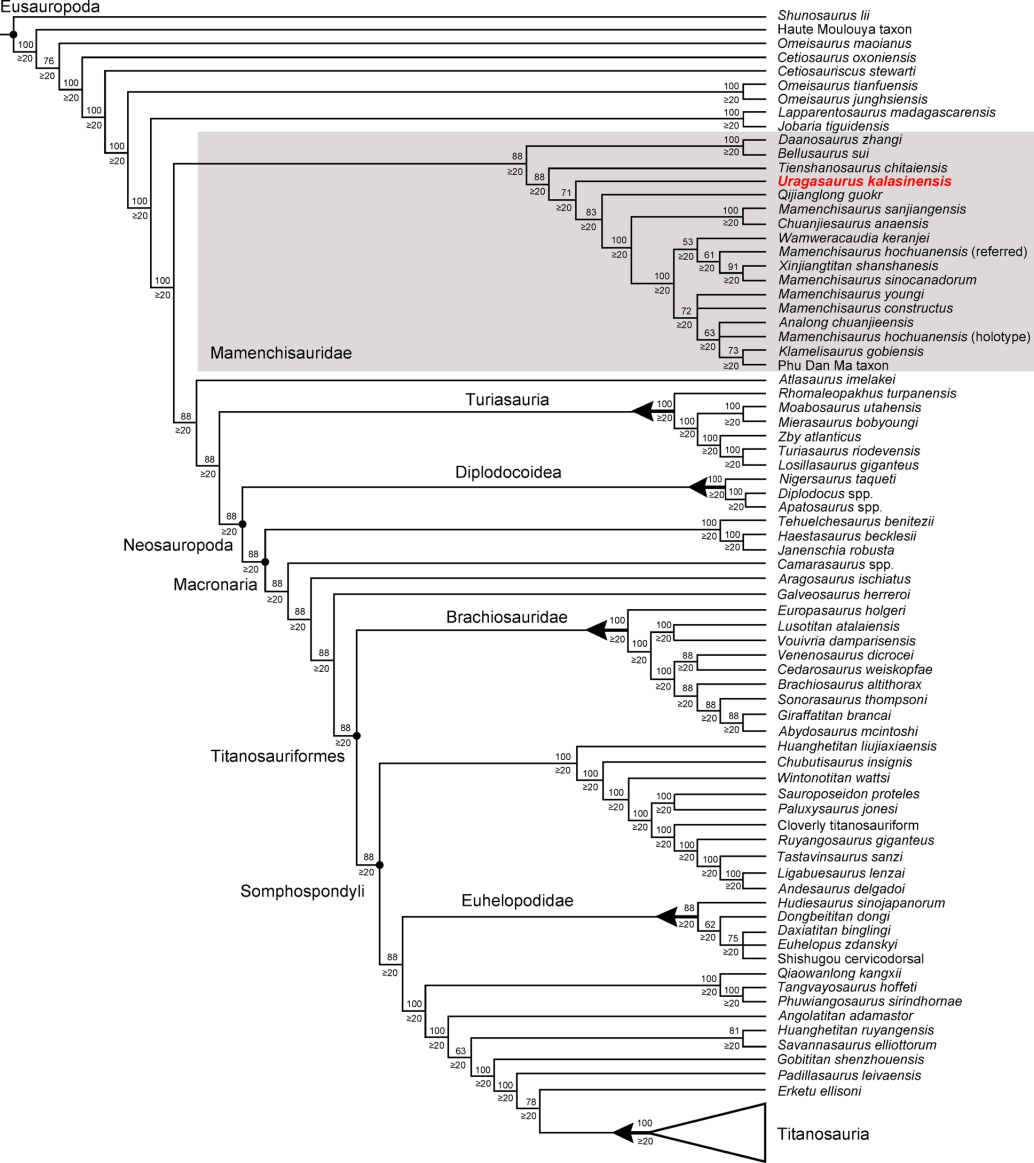
Phylogenetic result of the EIW analysis. 50% majority-rule consensus of the 500,000 MPTs (Tree length = 2084 steps) showing the phylogenetic position of *Uragasaurus kalasinensis* gen. et sp. nov. The number under each node indicates the the percentage frequency of clades recovered among the MPTs. The number under each node indicates the Bremer support value, which is applicable when the value is more than one. Consistency index (CI) = 0.232, retention index (RI) = 0.602.

The phylogenetic analysis is based on the data matrix of Moore et al.^11^, which was modified from Mannion et al.^30^, Moore et al.^25^, and Upchurch et al.^57^. The analysis under implied weighting recovered 500,000 most parsimonious trees (MPTs) of 2152 steps (CI = 0.224, RI = 0.585). The strict consensus tree is poorly resolved due to the presence of wildcard taxa, but the reduced strict consensus and 50% majority-rule trees show largely congruent topologies (Fig. 5). Moreover, the clade Mamenchisauridae, the node including *Daanosaurus zhangi* to Phu Dan Ma taxon, is resolved as monophyletic and supported by several synapomorphies: (1) the lateral pneumatic foramina of the anterior dorsal centra has acute posterior margin (Ch 146:1); (2) the middle to posterior dorsal neural arches have a narrow hyposphene (Ch 150:0); (3) the presence of the dorsal platform of the sacral series by the fusion of the first or fourth sacral neural spines (Ch 174:1); (4) the middle caudal vertebrae have anteroposteriorly widen base of the neural spines (Ch 199:1); (5) scapular blade has subtriangular process at anteroventral corner (Ch 216:1); (6) the ratio of maximum mediolateral width of distal end to proximodistal length of the humerus is 3.0 or greater (Ch 370:0); and (7) prdl of the middle and posterior cervical vertebrae have convex or with distinct bulging interruption in lateral view (Ch 434:1).

In all optimal trees, *Uragasaurus kalasinensis* is recovered within Mamenchisauridae, as an early-diverging lineage, positioned basal to a clade comprising *Qijianglong* and more derived mamenchisaurids. The node uniting *Tienchanosaurus*, *Uragasaurus*, and the more derived members of the clade is supported by three synapomorphies: (1) maximum to minimum dorsoventral height ratio of the scapular blade is less than 2.0 (Ch 37:1); (2) parapophysis of the postaxial cervical centra are dorsally excavated (Ch 121:1); and (3) postaxial cervical and anterior dorsal neural spines are bifurcated (Ch 132:1). Bremer support values along the backbone of Mamenchisauridae are generally moderate to high (≥ 20), indicating relatively stable relationships within the clade. However, the precise placement of the new taxon remains weakly supported, likely reflecting limited anatomical overlap and the high proportion of missing data for the taxon. This phylogenetic placement is consistent with the combination of plesiomorphic and derived characters observed in the anterior dorsal vertebra of *Uragasaurus*.

Wildcard taxa identified during the analysis include *Uragasaurus*, *Wamweracaudia*, *M. constructus*, *Analong*, *Abydosaurus*, *Dongbeititan*, *Angolatitan*, and *Epachthosaurus*, the removal of which substantially improves resolution without altering the placement of the new taxon. The unstable behaviour of *Uragasaurus* likely reflects the limited anatomical overlap with many taxa in the dataset and the presence of missing data, which reduces the number of characters available to constrain its placement. In addition, the combination of plesiomorphic and derived features results in character conflict among early-diverging mamenchisaurids, further contributing to its wildcard behaviour. No unambiguous synapomorphies were recovered for *Uragasaurus kalasinensis*, reflecting the fragmentary nature of the material and the high degree of character conflict within early-branching eusauropods.

## Discussion

*Uragasaurus kalasinensis* gen. et sp. nov. is recovered within Mamenchisauridae in the phylogenetic analyses and is positioned near the base of the clade, forming an early-diverging lineage basal to the clade comprising *Qijianglong* and more derived mamenchisaurids. However, the precise phylogenetic placement of the new taxon remains weakly supported. This instability likely reflects the limited anatomical overlap with many taxa in the dataset and the high proportion of missing data, which reduces the number of characters available to constrain its placement. Several vertebral characters used in mamenchisaurid phylogenetic analyses exhibit homoplastic distributions among eusauropods, particularly features related to pneumatic structures and laminar configurations. Such homoplasy likely reflects convergent functional or structural adaptations in sauropod vertebrae. Despite this, the phylogenetic analyses consistently recover *Uragasaurus kalasinensis* within Mamenchisauridae, suggesting that its placement is supported by the combined phylogenetic signal of multiple characters rather than any single feature.

The Y-shaped configuration formed by the stprl and tprl appears to be unique among currently known mamenchisaurids, although a superficially similar condition occurs independently in *Europasaurus holgeri*^55^. The combination of morphological characters observed in *Uragasaurus* indicates that several features of the new taxon exhibit homoplasy with other eusauropod and neosauropod lineages^7–10^. In addition, the camellate internal pneumatic structure within the centrum of *Uragasaurus*, confirmed by CT data, is a condition commonly reported in mamenchisaurids but also occurs convergently in several other eusauropod lineages. Similar camellate internal structures are present in some Asian eusauropods traditionally referred to Mamenchisauridae, such as the postaxial cervical vertebrae of *Omeisaurus tianfuensis*, which show numerous small-ellipsoid shape cavities on the eroded articular condyle^16^, as well as in derived titanosauriform clades, including Euhelopodidae and Titanosauria^7–10,52,53^. These patterns are consistent with previous studies suggesting considerable morphological convergence among early-branching eusauropods and derived titanosauriform sauropods.

**Fig. 6:**
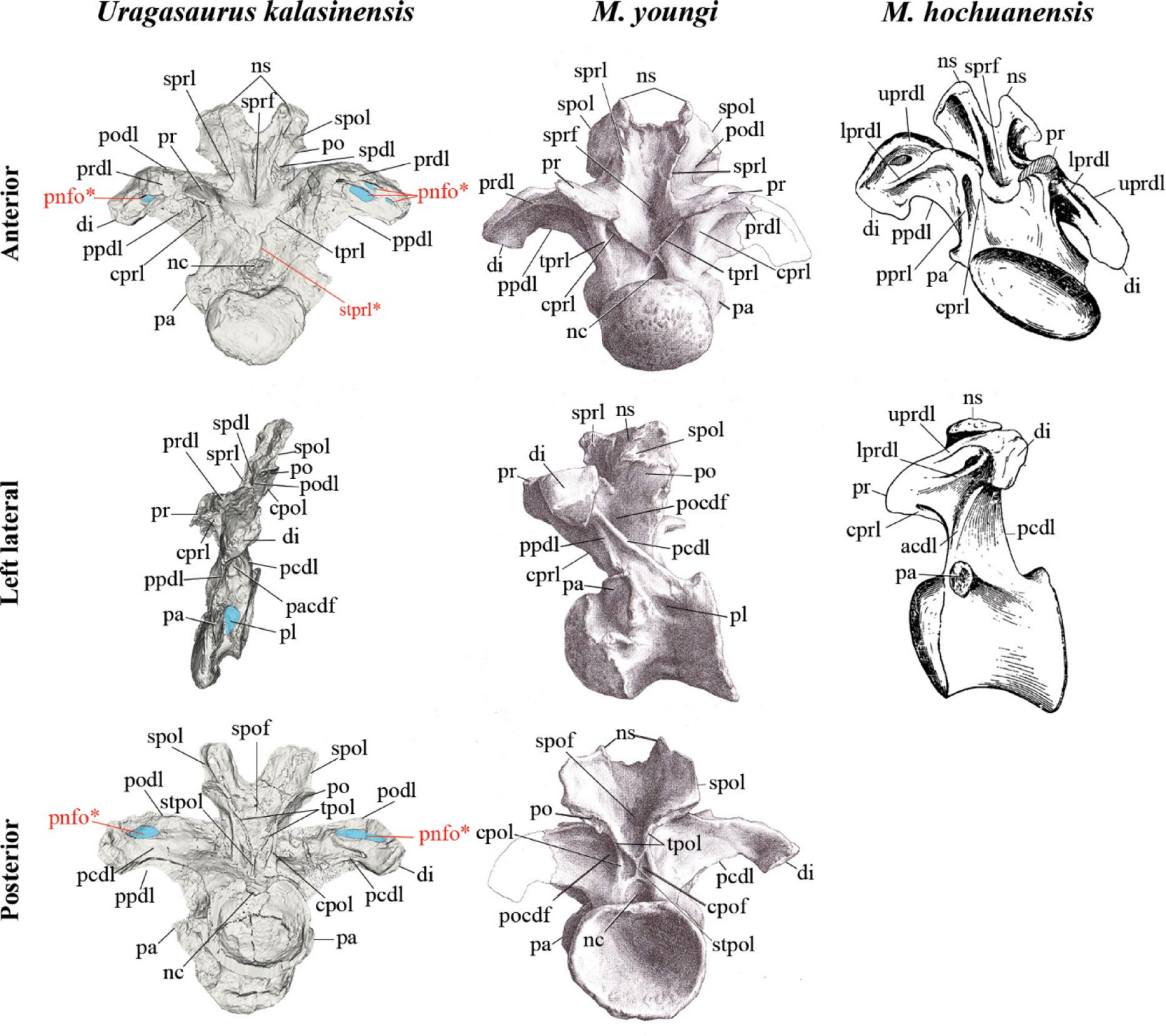
The comparison of anterior dorsal vertebrae of *Uragasaurus kalasinensis* with *M. youngi and M*. *hochuanensis* (modified from^1,14^. *Abbreviations* lprdl, lower prezygodiapophyseal lamina; uprdl, upper prezygodiapophyseal lamina. Other abbreviations are in Fig. 3. Asterisk refers to an autapomorphic character. The blue highlight indicates the pneumatic fossa and pleurocoel. Not to scale.

*Uragasaurus* exhibits distinct pneumatic fossae on the anterior and posterior surfaces of the transverse process. In contrast, the transverse processes of *Klamelisaurus*, and *M. youngi* are comparatively smooth and lack of fossa^14,25^. On the other hand, *M. hochuanensis*, by comparison, possesses a single teardrop-shaped fossa, formed by the separation of the upper and lower prdl (uprdl and lprdl) rather than the three distinct fossae observed in *Uragasaurus*^1^ (Fig. 6). Alternatively, *Omeisaurus* spp. represent multiple fossae similar to the condition in *Uragasaurus*. However, the fossae are primarily developed on the neural spine rather than on the neural arch or transverse process^16,56^. This indicates that the presence of multiple pneumatic fossae in anterior dorsal vertebrae may represent an independently evolution feature within mamenchisaurids and closely related non-neosauropodan eusauropods.

*Uragasaurus* provides further evidence of the diversity and distribution of mamenchisaurid sauropods in the southeast Asia. Contemporaneous Late Jurassic Mamenchisaurids include taxa such as *Hudiesaurus sinojapanorum* from the Kalazha Formation of the Turpan Basin, northwestern China, and the African mamenchisaurid *Wamweracaudia* from Tanzania^22,30,57^. Although *Rhomaleopakhus turpanensis* has previously been referred to the family, our phylogenetic analyses consistently recover it outside the clade, within Turiasauria in both 50% majority-rule and reduced strict consensus trees (Fig. 5). This result suggests that some Late Jurassic Asian eusauropods previously assigned to Mamenchisauridae may require re-evaluation. Within the Lower part of the Phu Kradung Formation of the Indochina terrane, Thailand, *Uragasaurus kalasinensis* gen. et sp. nov. currently represents the only formally named taxon (this study).

The “Phu Dan Ma taxon” (KS26-4) from the Phu Dan Ma locality, Upper part of the Phu Kradung Formation, has been interpreted as a derived mamenchisaurid and cited as evidence supporting an Early Cretaceous (Berriasian) age for at least part of the Upper part of the Phu Kradung Formation^29^. However, this specimen represents a cervicodorsal element by the position of the parapophysis on the ventrolateral border of the centrum, whereas the holotype of *Uragasaurus kalasinensis* consists of an anterior dorsal vertebra. Because vertebral morphology changes substantially along the cervical-dorsal transition in sauropods, many anatomical characters cannot be directly compared between these positions in the vertebral column. In particular, features used to diagnose *Uragasaurus*, including the prominent pneumatic fossae on the distal region of the transverse process and the specific laminar configuration of the anterior dorsal neural arch, cannot be evaluated in the Phu Dan Ma specimen. Consequently, the currently available material does not allow a direct test of whether the autapomorphy of *Uragasaurus* is present in KS26-4. Although the Phu Dan Ma taxon may also represent a mamenchisaurid sauropod, the lack of overlapping anatomical elements prevents confident referral of that specimen to *Uragasaurus* at present. It is therefore possible that the Phu Dan Ma specimen represents a different taxon or a distinct anatomical element of a related sauropod. However, the currently available material does not provide sufficient overlapping characters to test this hypothesis. *Uragasaurus* occurs in strata generally regarded as Late Jurassic, supporting the interpretation that the Phu Kradung Formation spans a stratigraphically extended interval from the Late Jurassic into the earliest Cretaceous.

**Fig. 7:**
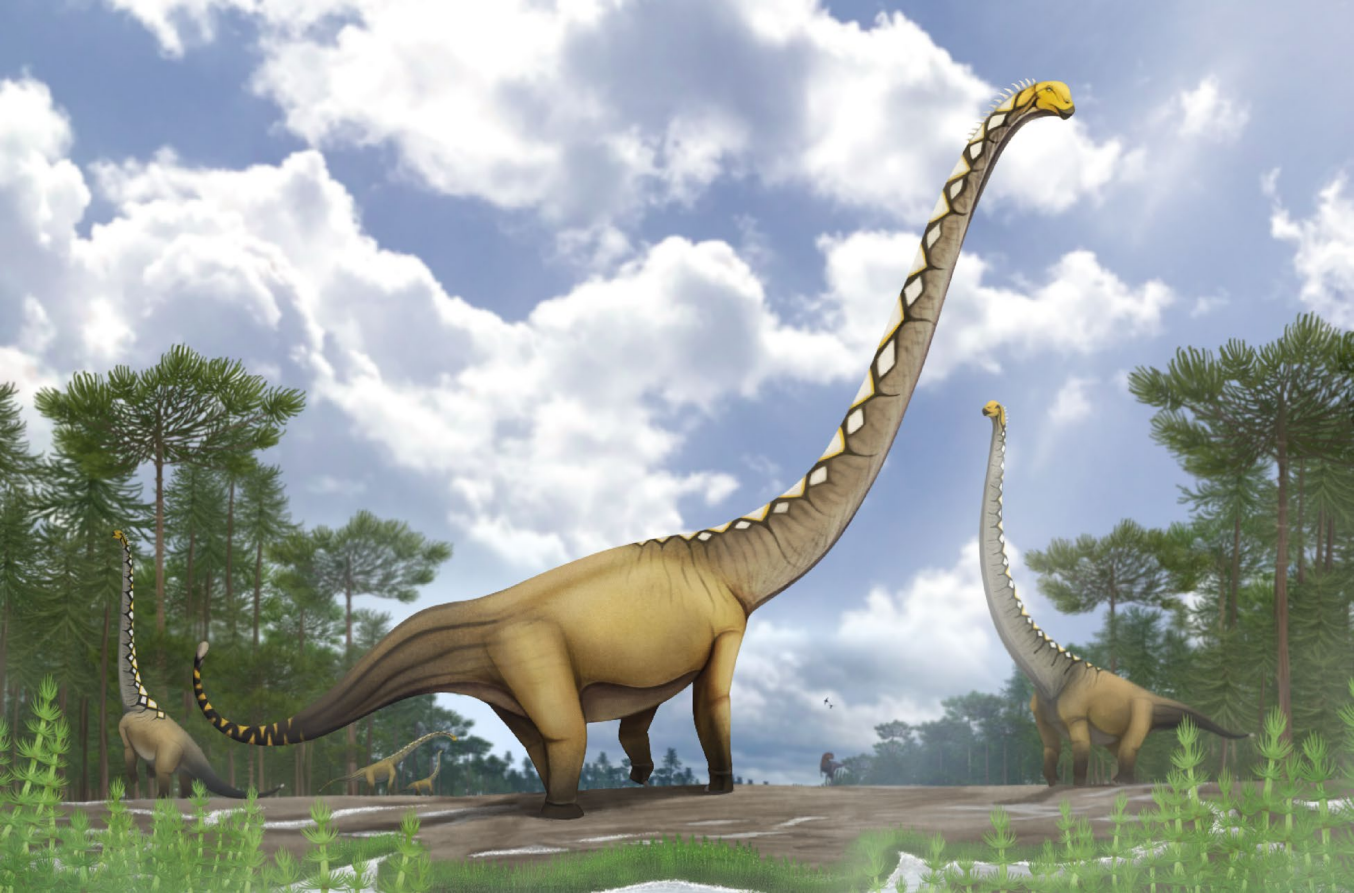
Life reconstruction of a herd of five individuals of *Uragasaurus kalasinensis* inhabiting a Late Jurassic forest in Thailand, accompanied by a pair of rhamphorhynchoid pterosaurs and a metriacanthosaurid theropod. Artwork by Pakorn Chotchaiyaporn (Jæsica ẞababi).

The distribution of sauropods across the Phu Kradung Formation contributes to broader discussions of vertebrate faunal change between the Late Jurassic assemblages of the Lower part of the Phu Kradung Formation and the Early Cretaceous assemblages of the Upper part of the Phu Kradung Formation (e.g., hybodont sharks, turtles, and crocodyliforms^38–40^). However, rather than indicating a sharp Jurassic-Cretaceous boundary turnover, the occurrence of *Uragasaurus* in the Lower part of Phu Kradung Formation is consistent with regional diversification during the Late Jurassic, preceding the more pronounced faunal reconstruction suggested for the Early Cretaceous. The currently limited theropod and ornithischian record from the Upper part of the Phu Kradung Formation constraints more detailed evaluation of dinosaurian faunal patterns^29^. The Phu Noi Locality preserves a substantial assemblage of sauropod material, and future discoveries may further clarify patterns of diversity and temporal distribution in this region (Fig. 7).

The discovery of *Uragasaurus kalasinensis* expands the geographic distribution of Mamenchisauridae into mainland Southeast Asia and provides additional evidence that this clade was widespread across eastern Asia during the Late Jurassic. The presence of closely related taxa in China suggests possible faunal connections across the East Asian landmass during this time. Comparisons with more distantly distributed taxa, such as *Wamweracaudia* from Africa^30^, highlight the complex paleobiogeographic patterns of sauropods during the Jurassic–Cretaceous transition, although the limited fossil record currently prevents a detailed reconstruction of dispersal routes. The phylogenetic placement of *Uragasaurus* near the base of Mamenchisauridae further highlights the morphological variability present among early-diverging members of the clade. Continued discoveries from the Phu Kradung Formation and other Jurassic deposits in Southeast Asia may therefore provide important insights into the early evolutionary history and biogeographic dispersal of mamenchisaurid sauropods.

The apparent differences between vertebrate assemblages from the lower and upper parts of the Phu Kradung Formation may suggest a faunal transition within the formation. However, because this interpretation currently relies primarily on sauropod occurrences and previously reported vertebrate assemblages, further discoveries and more comprehensive analyses will be required to fully evaluate this pattern.

## Conclusion

*Uragasaurus kalasinensis* gen. et sp. nov. from the Phu Kradung Formation (Upper Jurassic) of northeastern Thailand represents the first formally named mamenchisaurid sauropod from the country. Phylogenetic analyses recover the new taxon as an early-diverging member of Mamenchisauridae, expanding the known diversity and geographic distribution of the clade in Southeast Asia. Its occurrence in the Lower part of Phu Kradung Formation also supports the interpretation that the lower part of the formation is Late Jurassic in age, whereas the upper part corresponds to the earliest Cretaceous.

## Methods

The specimen was described at the Palaeontological Research and Education Centre, Mahasarakham University, Thailand. Measurements of the specimen have been provided in Table 1. The dorsal vertebrae, the holotype, and two dorsal vertebrae of the locality (See Supplementary Data 1), are scanned using a Canon Aquilion Prime SP CT Scanner 135 kVp. The resulting 3D models were reconstructed from 1023 slices per axis (X, Y, and Z) using the open-source Slicer 5.6.2 software (https://www.slicer.org/)^58^.

The phylogenetic relationships of *Uragasaurus kalasinensis* were assessed using a modified morphological character–taxon matrix of Moore et al.^11^, which follows the dataset lineage of Carballido et al.^59^, González Riga et al.^60^, Moore et al.^25^, and Upchurch et al.^57^. The matrix was edited in the Mesquite software^62^ and supplemented with additional taxa and revised character scorings based on firsthand observations and computed tomography (CT) data. *Uragasaurus kalasinensis* was coded for 10 characters, primarily dorsal vertebral characters (Supplementary Table S1). To reduce the influence of highly homoplastic characters, we conducted an additional analysis using extended implied weighting (EIW). The final phylogenetic matrix used in the analyses is analyzed in the Tree analysis using New Technology software (TNT)^61^. Both data matrices in the .nex and .tnt files are provided as Supplementary Data.

The initial dataset comprised 113 taxa, including the recently described *Mamenchisaurus sanjiangensis* and *Uragasaurus kalasinensis*, and 449 morphological characters. Characters 11, 14, 15, 27, 40, 51, 104, 122, 147, 148, 195, 205, 259, 297, 430, 438, and 449 were treated as ordered, following the treatment established in the original dataset of Moore et al.^11^. The characters 14, 20, 122, 130, and 258 were set as inactive. These characters were reformulated in previous revisions of the dataset: characters 14, 20, 122, and 130 were replaced by characters 430, 438, 431, and 433, respectively. Moore et al.^25^, and character 258 was reformulated as character 449^11^. The original versions were therefore excluded from the active character set to avoid redundancy while maintaining comparability with earlier iterations of the matrix.

Nine unstable operational taxonomic units (OTUs)—*Astrophocaudia*, *Australodocus*, *Brontomerus*, *Fukuititan*, *Fusuisaurus*, *Liubangosaurus*, *Malarguesaurus*, *Mongolosaurus*, and *Tendaguria*—were excluded prior to analysis. Node support was evaluated using Bremer support values. Additionally, the operational unit previously referred to as the “Phu Kradung taxon” was renamed the “Phu Dan Ma taxon” to avoid confusion with the new taxon described herein, as both derive from the Phu Kradung Formation.

The final matrix comprised 104 taxa and 449 characters and was analyzed under EIW with a concavity constant (K) of 12, following the protocol adopted in recent analyses of this dataset (Moore et al.^11,25^). Simulation studies have shown that implied weighting with k ≈ 12 can outperform both equal-weights parsimony and analyses using lower k values, which more strongly penalize homoplastic characters (Goloboff et al.^61^). Consequently, using K = 12 is a balanced, empirically supported parameter choice that has been shown to produce stable, biologically realistic topologies in large morphological datasets. Accordingly, this value was chosen as a balanced and empirically supported parameter setting.

Phylogenetic analyses were conducted in TNT version 1.6. Analyses were performed with 4000 MB of RAM allocated (*mxram* = *4000*) and a maximum tree buffer of 500,000 trees (hold 500,000). The collapsing rule followed TNT rule 3 (*maximum length* = *0*). A New Technology Search was conducted using 50 initial search replicates, requiring the best score to be hit 10 times, with default settings for sectorial searches, ratchet (five iterations), and tree fusing (*xmult* = *replications 50 hits 10 css rss ratchet 5 fuse 5*).

Subsequently, Tree Bisection–Reconnection (TBR) branch swapping was performed using a traditional search with 10 Wagner tree replicates, holding 10 trees per replicate. When a memory overflow occurred, an additional TBR search was conducted using trees retained in RAM.

Unstable OTUs were identified using the *pcrprune* and *nelsen* commands, and wildcard taxa were excluded using the “prune taxa” function within the tree buffer. Consensus topologies were summarised using reduced strict consensus and 50% majority-rule trees derived from all of the most parsimonious trees (MPTs). Tree length was calculated using the TNT tree score function, whereas the consistency index (CI) and retention index (RI) were computed using the *stats.run* script. Bremer support values were calculated using the TNT Bremer support function with a suboptimal search limit of 10 steps. 

### Nomenclature acts

This published work and the nomenclatural acts it contains have been registered in ZooBank. The ZooBank LSID for this publication is: LSIDurn: lsid.org: pub:951E978A-8450-4C4B-96CD-B2E625FD49BF.

## Supplementary Information

Below is the link to the electronic supplementary material.

Supplementary Material 1

Supplementary Material 2

Supplementary Material 3

Supplementary Material 4

Supplementary Material 5

## Data Availability

The final phylogenetic matrix used in this study is provided as Supplementary Data in both .nex and .tnt formats. Descriptions of associated sauropod materials, a table of data matrix characters, and the reduced strict and strict consensus trees are provided in the Supplementary Information document. The three-dimensional digital model of the holotype specimen (PRC 460) is available in the Figshare repository at 10.6084/m9.figshare.31456219 and will be made available in a public repository upon publication. All other data generated or analyzed during this study are included in this published article and its supplementary information files.
