# Supplementary figures and images for: A new mamenchisaurid sauropod from the Lower Phu Kradung Formation, Upper Jurassic of northeastern Thailand
Source: Sci Rep. 2026 Jul 8;16:21205. doi: 10.1038/s41598-026-49822-3 (PMC13346820; doi:10.1038/s41598-026-49822-3)

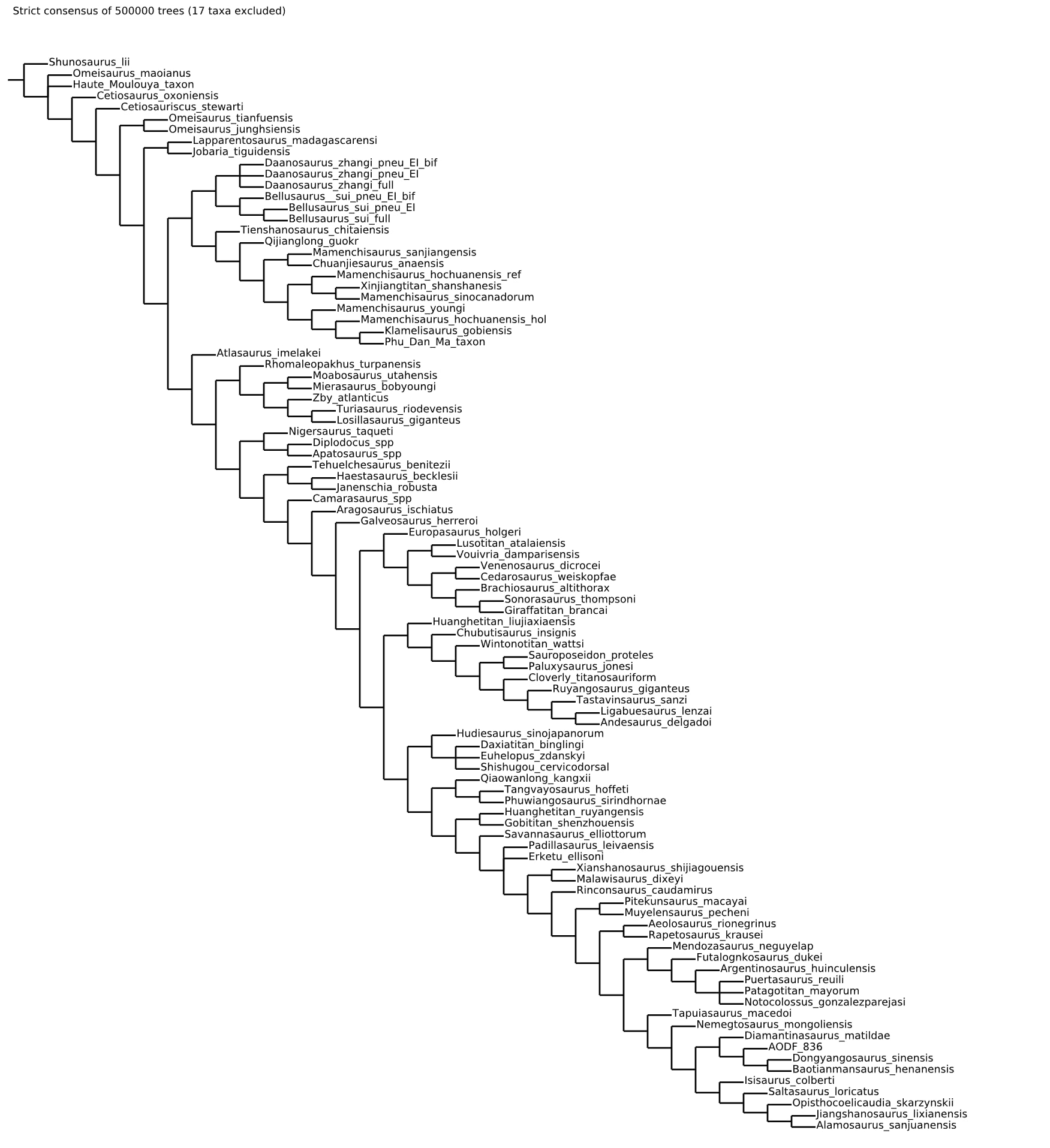

Supplement: Supplementary file 3 — Supplementary Material 3 [file 41598_2026_49822_MOESM3_ESM.jpg]

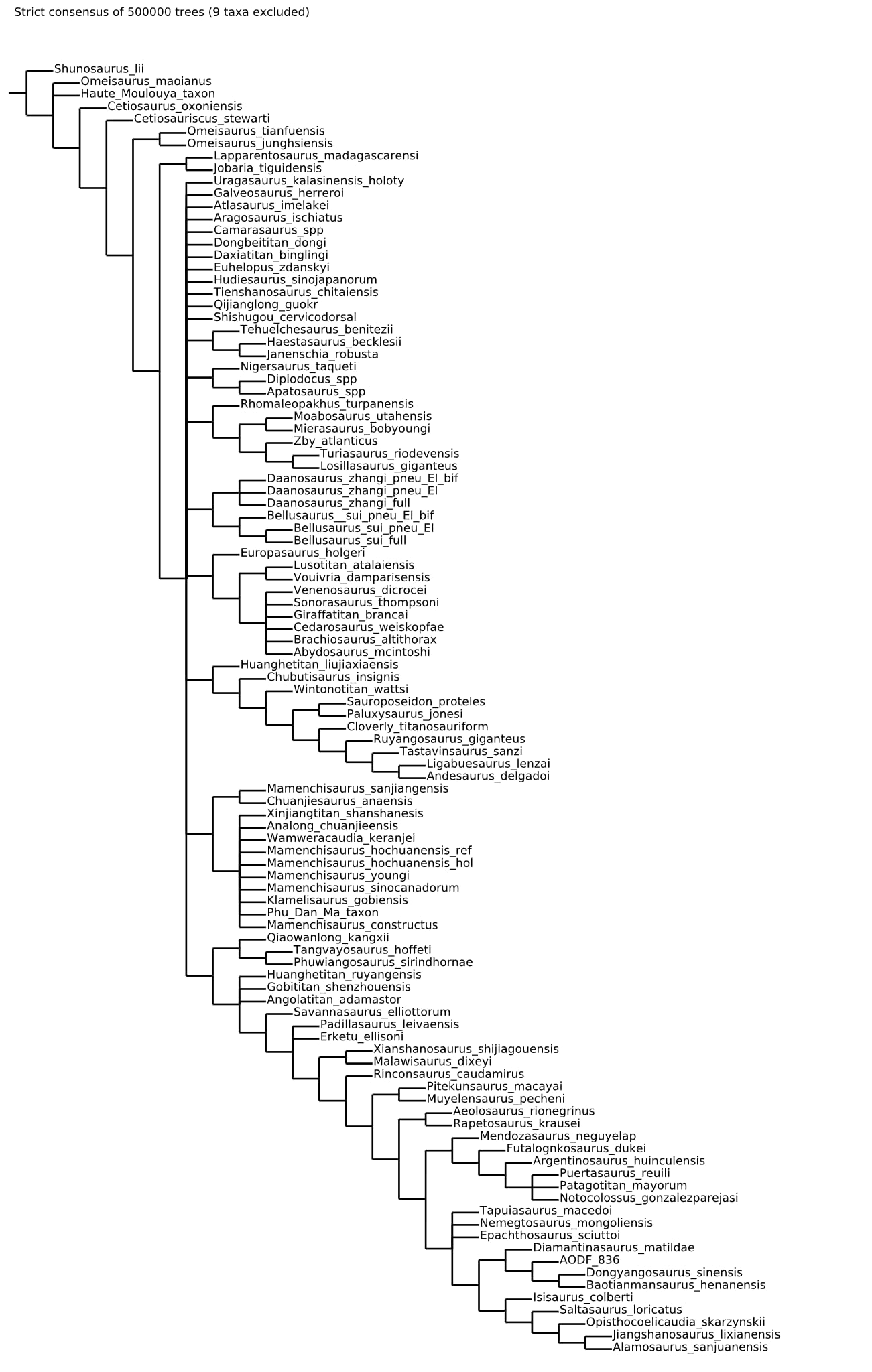

Supplement: Supplementary file 4 — Supplementary Material 4 [file 41598_2026_49822_MOESM4_ESM.jpg]
