# Supplementary material for: A new mamenchisaurid sauropod from the Lower Phu Kradung Formation, Upper Jurassic of northeastern Thailand
Source: Sci Rep. 2026 Jul 8;16:21205. doi: 10.1038/s41598-026-49822-3 (PMC13346820; doi:10.1038/s41598-026-49822-3)
Supplement: Supplementary file 5 — Supplementary Material 5 [file 41598_2026_49822_MOESM5_ESM.docx]

**Supplementary Information**

**A new mamenchisaurid sauropod from the Lower Phu Kradung Formation,
Upper Jurassic of northeastern Thailand**

***Apirut Nilpanapan^1^, Sita Manitkoon^2, 3^, Varavudh Suteethorn^4^, **Komsorn Lauprasert^1, 3^**

^1^Department of Biology, Faculty of Science, Mahasarakham University, Khamrieng, Maha Sarakham 44150, Thailand. ^2^Palaeontological Research and Education Centre, Mahasarakham University, Khamrieng, Maha Sarakham, 44150, Thailand, ^3^Vertebrate Palaeontology and Evolution Research Unit, Excellence Centre in Evolution of Life, Basin Studies and Applied Palaeontology, Mahasarakham University, Khamrieng, Maha Sarakham 44150, Thailand. ^4^Khon Kaen Geopark Association, Khon Kaen 40150, Thailand.

*First author: Apirut Nilpanapan (Email Address: Apirut.N@hotmail.com)
**Corresponding author: Komsorn Lauprasert (Email Address: komsorn.l@msu.ac.th)

**Supplementary Table S1.** Characters coded for *Uragasaurus kalasinensis*.

| **Character No.** | **Character State** |
| --- | --- |
| 115 | 2 |
| 143 | 1 |
| 144 | 0 |
| 145 | 1 |
| 153 | 0 |
| 154 | 0 |
| 160 | 0 |
| 332 | 0 |
| 336 | 1 |
| 337 | 0 |

**Supplementary Anatomical Abbreviations**

For vertebral laminae and fossae, we follow the anatomical terminology by Wilson (1999)^[1]^, Wilson et al. (2011)^[2]^, and Wilson Mantilla (2012)^[3]^: ap, aliform process; acpl, anterior centroparapophyseal lamina; acsl, accessory lamina; cod, condyle; cot, cotyle; cpol, centropostzygapophyseal lamina; cprl, centroprezygapophyseal lamina; di, diapophysis; hpo, hyposphene; hyp, hypantrum; nc, neural canal; ns, neural spine; pa, parapophysis; pcdl, posterior centrodiapophyseal lamina; pl, pleurocoel; pnf, pneumatic fossa; po, postzygapophysis; podl, postzygodiapophyseal lamina; posl, postspinal lamina; ppdl, paradiapophyseal lamina; pr, prezygapophysis; prdl, prezygodiapophyseal lamina; prpadf, prezygoparadiapophyseal fossa; prsf, prespinal fossa; spdl, spinodiapophyseal lamina; spof, spinopostzygapophyseal fossa; spol, spinopostzygapophyseal lamina; sprl, spinoprezygapophyseal lamina; td, transverse depression; tpol, intrapostzygapophyseal lamina; tprl, intraprezygapophyseal lamina.

**Supplementary descriptions**

**Associated sauropod materials**

**KS 34-581 dorsal neural arch**

A poorly preserved fragment of an anterior dorsal neural arch is more heavily anteroposteriorly compressed than the holotype PRC 460, and the distal half of the neural spine is chipped and broken (fig. S1a). As a result, the presence or absence of the neural spine bifurcation and the prespinal and postspinal laminae cannot be reliably assessed. Even though the transverse process has pneumatopores that resemble those of the holotype in shape and position, there is no physical match between the break-connecting surfaces. Despite the specimen being regarded as a possible holotype-associated fragment, it is not considered part of the holotype and is not used in the diagnosis.

**KS 34-586 dorsal neural arch**

The isolated neural arch, which lacks a paired centrum in both the bone map and the collection, is the closest specimen to the holotype to the south. The specimen is heavily compressed in the anteroposterior direction, with the distal end of fibula KS 34-588 attached to the proximal half of the neural spine area on the anterior surface, over prezygapophyses (fig. S1c). The position of the parapophysis is at the middle height of the neural arch, lower than the prezygapophysis, and the ventral margin is equal to the height of the dorsal edge of the neural canal. This can indicate the neural arch in the anterior dorsal position, corresponding to the third dorsal vertebrae of *O. tianfuensis*, *M. hochuanensis*, and *M. youngi*^[4-6]^. The ventral articulation surfaces display the typical crack lines of the bone matrix, along with several small, subcircular pneumatic cavities. These features indicate complete fusion between the neural arch and centrum prior to their separation. This evidence suggests that the specimen is in the adult stage, in contrast to the rugose, wrinkled surfaces of the unfused vertebrae found in juvenile specimens. For example, the anterior dorsal neural arch of the unnamed early juvenile valcanodontid from the Middle Jurassic of Chaiyaphum Province, Northeastern Thailand^[7]^, as well as the immature dorsal centra and neural arches of *Europasaurus holgeri*, and *Rapetosaurus krausei*^[8,9]^.

Furthermore, KS 34-586 represents an anterior dorsal neural arch with a single, non-bifid, triangular neural spine with well-developed, robust postspinal laminae on the anterior and posterior surfaces, respectively. This morphology contrasts with the dorsally cleft, bifid neural spine condition, which is absent of vertical laminae on the neural spine, present in the holotype. Overall, this specimen closely resembles the third dorsal vertebra of *O. tianfuensis* (illustrated in the published monograph^[4]^), except for the prespinal lamina, which has no illustration of the anterior surface, differing from the bifid neural spine condition present in the holotype and typical of mamenchisaurid-like taxa (*sensu* Moore et al., 2020^[10]^). Accordingly, KS 34-586 is not referred to the new taxon *Uragasaurus kalasinensis* but is regarded as an additional *Omeisaurus*-like eusauropod morphotype within the assemblage, indicating the possible presence of more than one sauropod taxon at the locality. Additionally, the dorsal vertebrae of *O. tienfuensis* and many *Mamenchisaurus* spp. should be redescribed to enable a clearer comparison.

**KS 34-587 indeterminate laterality coracoid**

The incomplete coracoid fragment, which cannot be determined as the left or right side by the compression and erosion, is excavated in close proximity to the holotype and regarded as associated sauropod material (Sauropoda indet.) and not referable to the new taxon. The specimen shows typical characteristics of a sauropod, such as a sub-oval outline, an increase in convexity and thickness of the bone forward to the posterior scapular facet, and the coracoid fossa at the middle posterior area that penetrates the surface of the bone.

**KS 34-588 left fibula**

The specimen consists of fragments of the fibular shaft, including a portion physically attached to KS 34-586 and an additional isolated shaft with a proximal head, both in one piece, of a similar field number and relative size (fig. S1c). These elements likely represent disarticulated portions of the same fibula, which is difficult to diagnose by lateral compression.

In both the lateral and medial views, the proximal portion of the fibula appears as a broad structure with a gently concave anterior outline and a pronounced sigmoid posterior outline, as in *Chuanjiesaurus* and *M. hochuanensis*^[5,11]^. The anterior shaft has a smoothly convex surface, while the posterior outline is sharp and features a thin bulge that becomes straight toward the middle of the shaft. The lateral surface of the proximal half of the bone is slightly convex, while the medial surface is slightly concave, exhibiting a longitudinal crack near the anterior edge that extends toward the vertical sulcus along the middle of the shaft, similar to *M. hochuanensis*^[5]^.

The proximal head is flattened and gradually becomes more robust towards the distal head. It is strongly expanded from the proximal edge, then slightly tapers to form a plain surface at the middle of the shaft. The remaining part of the proximal head is slightly more convex on the anterior side and gradually flattens towards the posterior end.

The distal head is less expanded in the anteroposterior direction but is thicker in the mediolateral direction compared to the proximal head. The anterior outline of the distal head is straight, gradually curving to meet the shaft at the posterior outline. The lateral surface of the distal head is obscured by the dorsal vertebra, while the medial surface displays a rounded, convex distal head that connects to the lateral concave articular surface of the astragalus, like *Chaunjiesaurus*^[11]^.

**KS 34-602a middle cervical vertebra**

KS 34-602a (fig. S1b) is determined to be a middle cervical vertebra based on overall morphology, which is compared with that of other mamenchisaurids (see below). Because there are no other cervical vertebrae in the area and the specimen is deformed by taphonomy, the precise serial position of the vertebrae cannot be determined.

The middle cervical vertebra KS 34-602 is elongated in the anteroposterior direction and exhibits significant deformities. The anterior portion has been crushed and displaced ventrally, redirecting the anterior elements (including the anterior condyle, neural canal, and prezygapophyses) to shift onto the ventral surface. As a result, the anterior condyle has lost its original convexity, making it impossible to calculate the elongation index (EI) reliably, and the width-to-length ratio is not within its normal range. The dorsal surface is compressed and attached to the shaft of the dorsal rib, covering the area of the neural spine. The subrectangular parapophyses are anteroposteriorly flattened, located on the ventrolateral margin of the anterior condyle of the centrum. The ventral surface of the centrum is mildly transversely concave and bears posterior centroparapophyseal laminae (pcpl) along the ventrolateral margins. The ventral midline keel is absent, like *Chuanjiesaurus*, *Klamelisaurus*, *M. constructus*, and *M. jingyanensis*^[10-13]^, but opposite to the postaxial cervical centra of *O. tianfuensis* and *Xinjiangtitan*^[4,14]^. Due to firm dorsoventral compression, the lateral surfaces of the centrum are flattened, preventing observation of the presence or morphology of pneumatic fossae. Although the specimen is not well-preserved, the ventral surface of the anterior portion of the neural arch resembles that of cervical 5th to 7th of *M. jingyanensis*^[13]^, which possesses numerous pneumatic cavities.

The neural arch is relatively low, as indicated by the short height of the centroprezygapophyseal laminae (cprl), which form short, robust, pillar-like structures. The articular surfaces of the prezygapophyses are broad and oriented approximately 45° to the horizontal plane, expanding laterally over the cprl. The intraprezygapophyseal laminae (tprl) extend medioventrally between the prezygapophyses and form the dorsal margin of the neural canal. Between the tprl, neural canal, and cprl lies a pair of prominent, large oval fossae centroprezygapophyseal fossae (cprf). Posterior to the tprl, a vertically oriented prespinal fossa is present, laterally bounded by the compressed spinoprezygapophyseal laminae (sprl) and the neural spine. The transverse processes are subtriangular in outline and curve posteriorly, which is formed by laterally expanded flanges of the prezygodiapophyseal laminae (prdl) and posterior centrodiapophyseal laminae (pcdl).

On the dorsal surface, an indeterminate cervical rib overlies the neural spine, contacting the centrum via a posteriorly concave articular surface. This rib obscures observation of a postspinal lamina (posl), if present. The postzygapophyses are heavily damaged: the left is absent, whereas the right is partially preserved but lacks its lateral surface. Nevertheless, prominent spinopostzygapophyseal laminae (spol) extend from the neural spine to the postzygapophyses, forming a subtriangular spol beneath the misaligned rib. Finally, the transverse processes exhibit smooth, ventrolaterally inclined surfaces, in contrast to the high pneumatopores on their ventral surfaces. Overall, KS 34-602a represents a relatively elongated cervical vertebra with a low neural arch and an unknown spine, which could be interpreted as a low, flat type, like the spine of typical mamenchisaurids.

**KS 34-602b right cervical rib**

The specimen is a cervical rib head with a moderately eroded surface, showing the proximal half of the anterior process and the proximal end of the distal shaft (fig. S1e). It features a nearly complete capitulum with an ellipsoid concave articular surface. On the other hand, the tuberculum is transversely flattened and anteroposteriorly widened, with a slit-like articular surface that is perpendicular to the capitulum. The dorsomedial surface has a concave longitudinal groove with a transverse ridge connecting to the capitulum and tuberculum, effectively separating the surface into anterior and posterior grooves. The anterior groove features two small, rounded pneumatopores, while the posterior trough contains a larger, single one. The pneumatic cavity is present in *Bellusaurus*, but only one large cavity on the posterior groove adjacent to the transverse ridge^[10,15]^.

**Additional dorsal vertebra materials in the locality**

**PN 692 middle to posterior dorsal vertebra and PN 13-23 posterior dorsal vertebrae**

PN 692 is the middle dorsal vertebra with migrating parapophysis to the middle height of the neural arch, transversely straight transverse process, and a single neural spine with a large prsl (fig. S2 and fig. S3). The morphology of the vertebra corresponding to the middle dorsal of *Analong chuanjieensis*, *Omeisaurus* spp., and *Mamenchisaurus* spp., which has explicit prsl. PN13-23 is the posterior dorsal with the presence of ppdl, similar to *M. youngi*, which possesses the lamina only in the posterior dorsal. Opposed to PN692, the vertebra lacks distinct prsl but has a rough infill cavity of sprf instead, which resembles the middle to posterior dorsal vertebrae of *Klamelisaurus*^[10]^.

In anterior and posterior views, the centrum has a higher-than-wide subcircular outline with a flattened opisthocoelous centrum. The surface of the anterior condyle is eroded, revealing the random pattern of subcircular cancellous internal pneumatic tubers. The exposed internal structure resembles the cervical pneumatization of *M. sinocanadorum*^[16]^ and the cervical and dorsal vertebrae of the Late Cretaceous titanosaur *Saltasaurus* *loricatus*^[17]^. On the other hand, the exposed internal architecture in Phu Dan Ma cervicodorsal (KS26−4) is a subtrapezoidal honeycomb shaped camellate^[18]^. The ventral surface is transversely convex without ventral keel and angular edge between the ventral and lateral surfaces. Due to the heavy anteroposterior compression, the details of the lateral pneumatic fossa are unknown.

PN13-23 is affected by the oblique lateral compression that reveals the details of the lateral surfaces of the specimen. This vertebra represents the ventral keel and lateral pneumatic fossa with pneumatic foramen inside the anteroventral area of the fossa. In the articulated vertebra series of *Klamelisaurus*, the presence of the ventral keel is independent in each dorsal vertebra^[10]^, which cannot be determined as a distinctive taxon. Moreover, the pneumatic foramen inside the pneumatic fossa is present on the middle to posterior dorsal vertebrae but absent in the anterior dorsal, which is related to the pulmonary protuberances of the lung^[19,20]^.

**Neural arch**

The parapophysis is located at the middle height of the neural arch, between the prezygapophysis's medial edge and the midline lamina's middle height. Like the anterior dorsal vertebra (PN581), the articular surface of the parapophysis is highly compressed in an anteroposterior direction, transforming the articular facet into flat laminate bone. Parapophysis connects to acpl ventrally and prpl dorsomedially, the anteriorly compressed oblique large buttress between prezygapophysis and parapophysis. Medially to the parapophysis is the pair of rounded fossae cprf, which is enveloped by prpl laterodorsally, tprl dorsomedially, and midline lamina medially. The fossa is dorsoventrally shallower from the deepest area below to prpl and tprl to the plane at the ventral tip of midline laminaabove the transversely wide ellipsoid shape neural canal.

On the posterior surface, the ventral area of the neural arch is damaged. However, the trace of laminae is still present. There are three main vertical laminae on the surface: 1) the vertical midline lamina between the neural canal and tpol, 2) a pair of dorsomedial buttresses cpol from the ventrolateral edge of the neural arch to the tpol on midline region, 3) the pcdl lines along the lateral edge of neural arch to the ventral edge of the transverse process, which terminated at the ventral end of diapophysis. Furthermore, the parapophysis is visible in posterior view below the curve of the transverse process.

The lateral surfaces are represented by PN13-223, which has acpl beneath the elliptical parapophysis at the middle height of the neural arch. Knob-like diapophysis is completely preserved on the distal end of the left transverse process. It has ppdl, which is presented on the posterior dorsal vertebrae of mamenchisaurids^[6,10,19]^. Ppdl is parallel to prdl and creates the large fossa on the anterior surface of the transverse process, which is located between prezygapophysis, parapophysis, and diapophysis.

On the anterior view, the prezygapophysis has a ventromedially directed articular facet, the outline of a transversely wide plain area. Between the facets is the transversely narrow gap of the hypantrum, which is connected to hyposphene of the preceding vertebra. From the ventromedial edge of the facet is the ventromedial curve lamina of tprl, which meets its counterpart at the midline and then merges with the ventral midline lamina to create the Y-shape lamina at the middle of the neural arch. Moreover, the prezygapophysis is laterally connected to the diapophysis by the weakly upward-curved prdl in the middle of the transverse process, and dorsally connects to the neural spine via sprl. The postzygapophysis on the posterior surface has a ventrolaterally directed articular facet, which fits to the prezygapophysis facet of the succeeding vertebra. It connects to diapophysis laterally by the podl and dorsally to the neural spine by spol along the lateral margin.

The transverse process is weakly dorsolaterally flared, with a convex outline at the distal end, in the diapophysis region. The process consists of three main layers: 1) the sturdy upper middle horizontal prdl, 2) the podl lines along the dorsal margin, and 3) the pcdl lines along the ventral margin. There are two horizontal elongated fossae: 1) the upper part transverse depression between prdl and podl, and 2) the pneumatic fossa between prdl and ppdl.

**Neural spine**

As in common sauropods, there is a single vertical neural spine on the middle and posterior dorsal vertebrae on the sagittal plane of the bone^[13,17,24]^. The prsl is present in PN692 but absent in PN13-23, contrasting to the ppdl, which is absent in the former but present in the latter vertebra.

PN13-23 shows the morphology of the lateral surfaces of the neural spine. It settles on the posterior half of the centrum and comprises sprl on the anterior margin, which ventrally bifurcates at the middle height of the spine and laterally divides to spdl. On the anterodistal and posterodistal margin of the spine, the sprl and spol forms the blunt and low-degree subtriangular aliform process, resembling *Klamelisaurus*, *M. hochuanensis*, and *M. youngi*^[5,6,10]^. The middle area of the lateral surface is dorsoventrally elongated depression of spdf, enveloped by sprl anteriorly and spol posteriorly.

**Supplementary References**

1. Wilson, J. A. A nomenclature for vertebral laminae in sauropods and other saurischian dinosaurs. *Journal of Vertebrate Paleontology* vol. 19 639–653 (1999).

2. Wilson, J. A., D’Emic, M. D., Ikejiri, T., Moacdieh, E. M. & Whitlock, J. A. A nomenclature for vertebral fossae in sauropods and other saurischian dinosaurs. *PLoS One* **6**, (2011).

3. Wilson Mantilla, J. New Vertebral Laminae and Patterns of Serial Variation in Vertebral Laminae of Sauropod Dinosaurs. *Contributions from the Museum of Paleontology, University of Michigan* **32**, 91–110 (2012).

4. He, X. L., Li, K. & Cai, K. The Middle Jurassic dinosaur fauna from Dashanpu, Zigong, Sichuan. Vol. IV. Sauropod Dinosaurs (2) *Omeisaurus tianfuensis*.  *Sichuan Publishing House of Science and Technology* **4**, (1988).

5. Young, C. C. & Zhao, X. J. *Mamenchisaurus, Institute of Vertebrate Paleontology and Paleoanthropology*. *Monograph Series I,* vol. 8 (Science Press (in Chinese), 1972).

6. Ouyang, H. & Ye, Y. *The First Mamenchisaurian Skeleton with Complete Skull: Mamenchisaurus Youngi*. (Sichuan Publishing House of Science and Technology, 2002).

7. Hanta, R., Sekiya, T., Shibata, M., Naksri, W. & Tucker, R. T. Hatchling and early juvenile of early sauropod from the early Middle Jurassic Nam Phong Formation, Chaiyaphum Province, northeastern Thailand. *Paleontol. Res.* 28, 222–239 (2023).

8. Carballido, J. L. & Sander, P. M. Postcranial axial skeleton of *Europasaurus holgeri* (Dinosauria, Sauropoda) from the Upper Jurassic of Germany: Implications for sauropod ontogeny and phylogenetic relationships of basal Macronaria. *J. Syst. Palaeontol.* **12**, 335–387 (2014).

9. Rogers, K. C. The postcranial osteology of *Rapetosaurus krausei* (Sauropoda: Titanosauria) from the Late Cretaceous of Madagascar. *J. Vertebr. Paleontol.* **29**, 1046–1086 (2009).

10. Moore, A. J., Upchurch, P., Barrett, P. M., Clark, J. M. & Xing, X. Osteology of *Klamelisaurus gobiensis* (Dinosauria, Eusauropoda) and the evolutionary history of Middle–Late Jurassic Chinese sauropods. *J. Syst. Palaeontol.* **18**, 1299–1393 (2020).

11. Sekiya, T. Re-examination of *Chuanjiesaurus anaensis* (Dinosauria: Sauropoda) from the Middle Jurassic Chuanjie Formation, Lufeng County, Yunnan Province, Southwest China. *Memoir of the Fukui Prefectural Dinosaur Museum* **10**, 1–54 (2011).

12. Young, C.C. On a new sauropod from Yiping, Szechuan, China. *Acta Sci. Sin.* **3**, 491–504 (1954).

13. He, X. *et al.* A new species of sauropod, *Mamenchisaurus anyuensis* sp. nov. *Geosciences (Basel).* 83–86 (1996).

14. Zhang, X.Q., Li, N., Xie, Y., Li, D.-Q. & Hai-Lu, Y. Redescription of the dorsal vertebrae of the mamenchisaurid sauropod *Xinjiangtitan shanshanesis* Wu et al. 2013. *Hist. Biol.* **32**, 803–822 (2020).

15. Dong, Z. M. Sauropoda from the Kelameili Region of the Junggar Basin, Xinjiang Autonomous Region. *Vert. PalAsiat.* 28:43-58 (1990).

16. Moore, A. J. *et al.* Re-assessment of the Late Jurassic eusauropod *Mamenchisaurus sinocanadorum* Russell and Zheng, 1993, and the evolution of exceptionally long necks in mamenchisaurids. *J. Syst. Palaeontol.* **21**, 2171818 (2023).

17. Powell, J. E. Osteología de Saltasaurus loricatus (Sauropoda-Titanosauridae) del Cretácico Superior argentino. In Los dinosaurios y su entorno biótico: II Curso de Paleontología, 10 a 12 de julio de 1990. *Instituto Juan de Valdés. Actas* (165-230) (1992).

18. Suteethorn, S., Loeuff, J. Le, Buffetaut, E., Suteethorn, V. & Wongko, K. First evidence of a mamenchisaurid dinosaur from the Upper Jurassic-Lower Cretaceous Phu Kradung Formation of Thailand. *Acta Palaeontol. Pol.* **58**, 459–469 (2013).

19. Russell, D. A. & Zheng, Z. A large mamenchisaurid from the Junggar Basin, Xinjiang, People’s Republic of China. *Can. J. Earth Sci.* **30**, 2082–2095 (1993).

20. O’Connor, P. M. Postcranial pneumaticity: An evaluation of soft-tissue influences on the postcranial skeleton and the reconstruction of pulmonary anatomy in archosaurs. *Journal of morphology* 267, 1199–1226 (2006).

**Supplementary Figures**


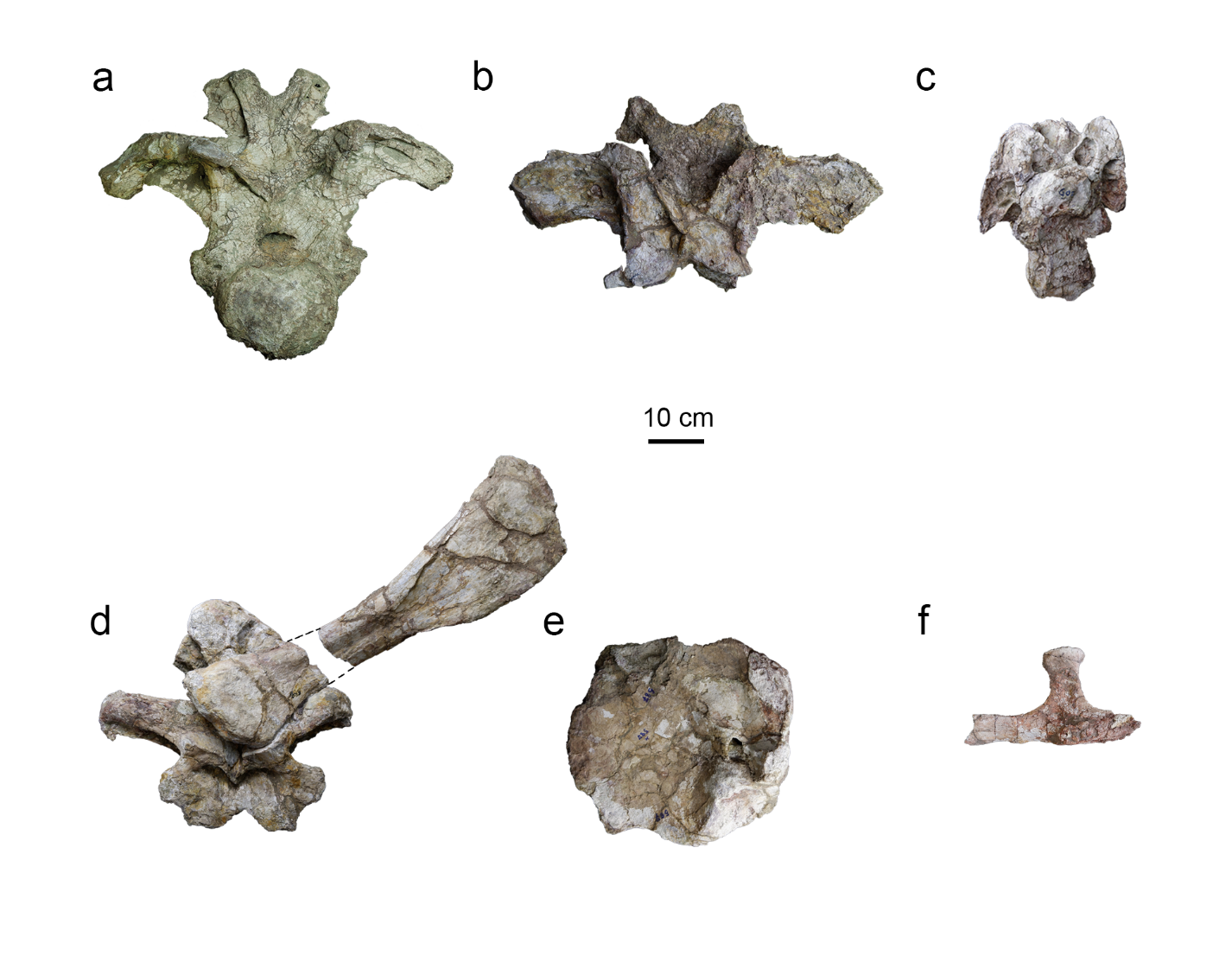


**Supplementary Fig. S1.** Associated sauropod materials from the Phu Noi locality. KS 34-581 dorsal neural arch (a); KS 34-602a cervical vertebra (b); KS 34-586 dorsal neural arch attached with KS 34-588 left fibula (c); KS 34-587 coracoid (d); KS 34-602b right cervical rib (e).


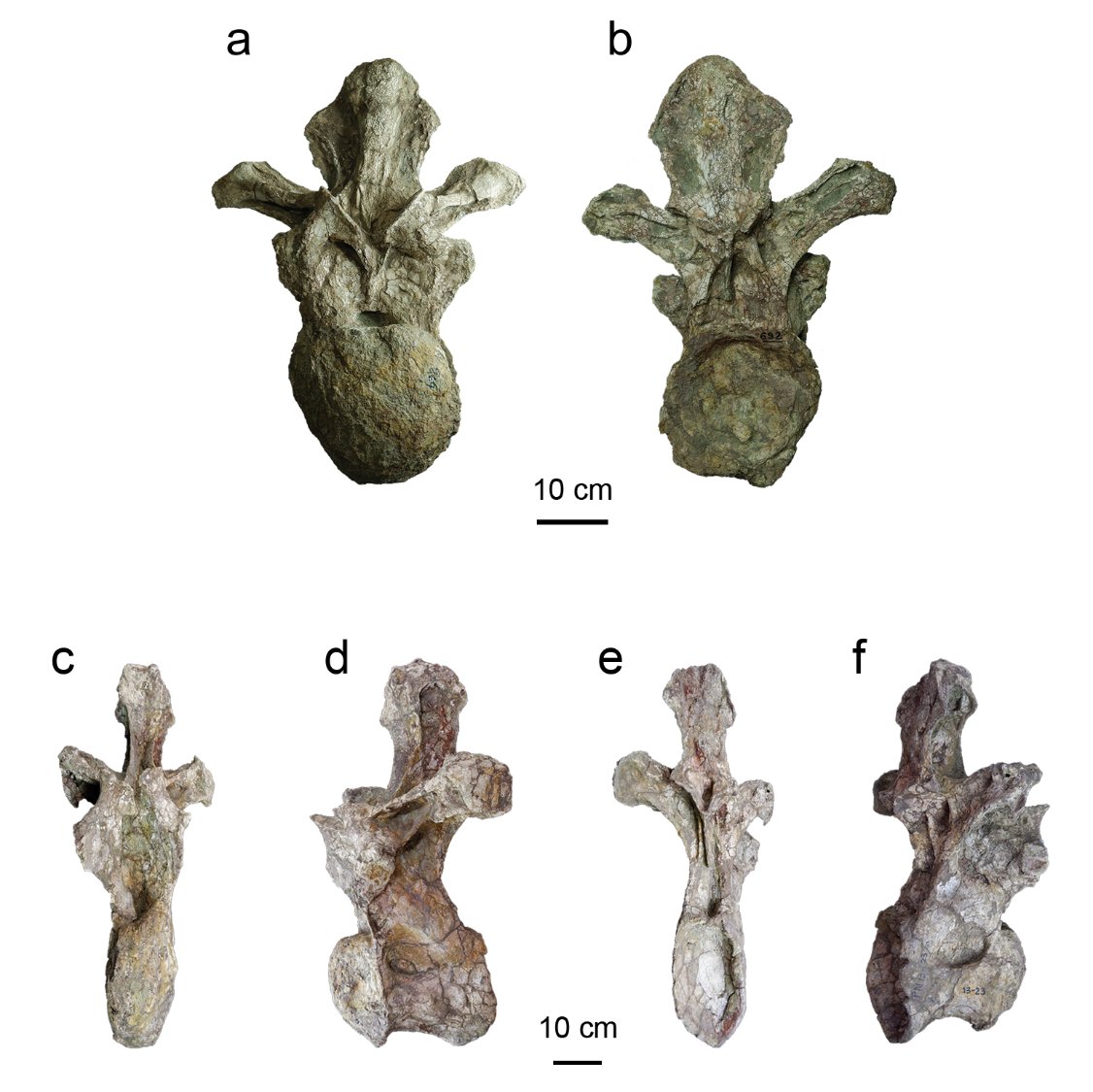


**Supplementary Fig. S2.** Associated sauropod dorsal vertebrae from the Phu Noi locality. Middle to posterior dorsal vertebra PN 692 in anterior (a) and posterior (b) views; Posterior dorsal vertebra PN 13-23 in anterior (c), left lateral (d), posterior (e), and right lateral (f) views.


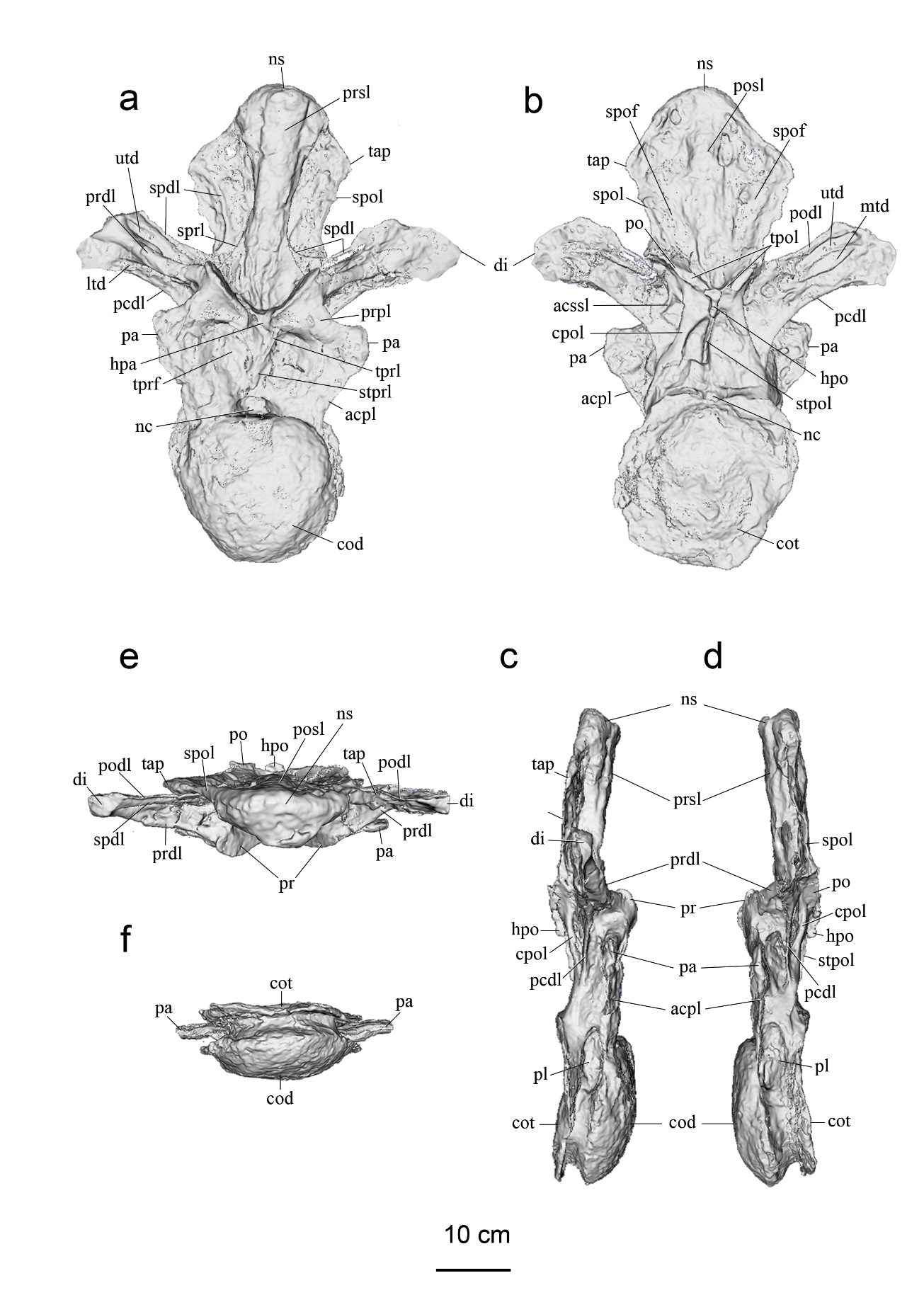


**Supplementary Fig. S3.** Digital rendering of PN 692 in anterior (a); posterior (b); right lateral (c); left lateral (d); dorsal (e); and ventral (f).


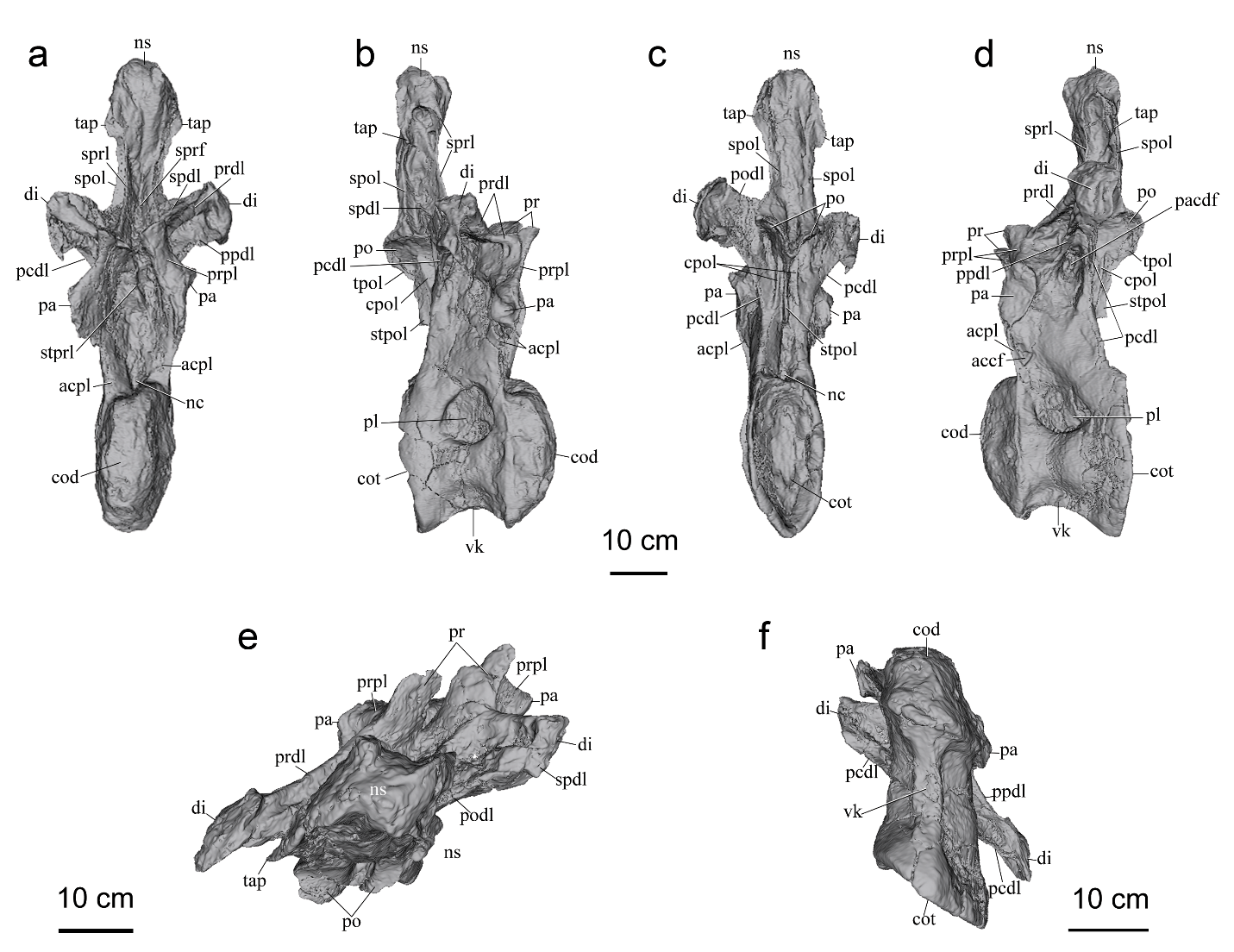


**Supplementary Fig. S4.** Digital rendering of PN 13-23 in anterior (a); posterior (b); right lateral (c); left lateral (d); dorsal (e); and ventral (f).
